# Malignancy pattern analysis of breast ultrasound images using clinical features and a graph convolutional network

**DOI:** 10.1177/20552076241251660

**Published:** 2024-05-15

**Authors:** Sidratul Montaha, Sami Azam, Md. Rahad Islam Bhuiyan, Sadia Sultana Chowa, Md. Saddam Hossain Mukta, Mirjam Jonkman

**Affiliations:** 1 Department of Computer Science, 152054 University of Calgary, Calgary, Canada; 2 Faculty of Science and Technology, 10095 Charles Darwin University, Casuarina, Australia; 3 130062 United International University (UIU) United City, Dhaka, Bangladesh

**Keywords:** Breast ultrasound image, graph convolutional network, clinical features, ensemble learning, graph attention network

## Abstract

**Objective:**

Early diagnosis of breast cancer can lead to effective treatment, possibly increase long-term survival rates, and improve quality of life. The objective of this study is to present an automated analysis and classification system for breast cancer using clinical markers such as tumor shape, orientation, margin, and surrounding tissue. The novelty and uniqueness of the study lie in the approach of considering medical features based on the diagnosis of radiologists.

**Methods:**

Using clinical markers, a graph is generated where each feature is represented by a node, and the connection between them is represented by an edge which is derived through Pearson's correlation method. A graph convolutional network (GCN) model is proposed to classify breast tumors into benign and malignant, using the graph data. Several statistical tests are performed to assess the importance of the proposed features. The performance of the proposed GCN model is improved by experimenting with different layer configurations and hyper-parameter settings.

**Results:**

Results show that the proposed model has a 98.73% test accuracy. The performance of the model is compared with a graph attention network, a one-dimensional convolutional neural network, and five transfer learning models, ten machine learning models, and three ensemble learning models. The performance of the model was further assessed with three supplementary breast cancer ultrasound image datasets, where the accuracies are 91.03%, 94.37%, and 89.62% for Dataset A, Dataset B, and Dataset C (combining Dataset A and Dataset B) respectively. Overfitting issues are assessed through k-fold cross-validation.

**Conclusion:**

Several variants are utilized to present a more rigorous and fair evaluation of our work, especially the importance of extracting clinically relevant features. Moreover, a GCN model using graph data can be a promising solution for an automated feature-based breast image classification system.

## Introduction

Since, breast cancer has become one of the most prevalent cancers worldwide, it leads to 0.68 million (6.7%) of all newly diagnosed cancer deaths and is the fifth most common cause of cancer deaths among women.^1^ The World Health Organization (WHO) estimates that 19.3 million new cases of cancer will be diagnosed worldwide in 2025.^2^ The typical age of diagnosis is between 45 and 55 years of age. Patients under the age of 35 comprise about 10 to 15% of the total number of cases.^3^ Breast cancer may not exhibit noticeable signs in the early stages, which can cause a delay in diagnosis and treatment. However, breast cancer can grow and spread if neglected, which makes it more challenging to control and cure. Early detection and treatment are key factors in improving the outcome and reducing the risk of complications associated with breast cancer. According to a recent study, early detection boosts 5-year survival rates to 91%.^4^ Breast cancer mortality in average-risk women can be reduced by 20% with routine screening, starting at age 40.^5^ One of the clinically preferred diagnostic techniques for breast cancer screening is ultrasonography which can show the inner structure (outline, edge), activity and blood flow of the breast tumors.^6^ However, distinguishing benign and malignant masses can be challenging for small lesions. Breast cancer clinical tests can be both costly and time-consuming. This can lead to delayed diagnosis and treatment, which can have serious consequences for patients’ health outcomes. Due to the complex structures of breast masses, radiologists need to devote considerable time and effort to diagnosing tumors. An additional problem is the global shortage of expert radiologists and medical professionals who can interpret the screening data, particularly in rural areas and poor nations.^7^ An automated system using ultrasound images may assist radiologists and can be a promising diagnostic solution for breast cancer remarkably.

In clinical practice, masses are distinguished based on their shape, alignment, borders, and contiguous tissue structures. The existence of calcifications shape and edge in particular, are the most significant diagnostic clinical features when assessing breast lesions. Extracting computerized features from ultrasound images according to clinical diagnostic markers can be an effective solution. By doing so, the radiologists’ methods can be mimicked by an automated approach. This research primarily focuses on extracting computerized features from the ultrasound images of benign and malignant lesions according to the clinical markers of breast cancer. Seven handcrafted features are extracted from the breast cancer ultrasound images based on tumor shape, margin, lateral dimension, and characteristics of surrounding tissues. These features are considered as Breast Imaging Reporting and Data System (BI-RADS) features and to the best of our knowledge no prior study found that incorporates these computerized features for automated classification with graph representation. This is a novel contribution as it employs graph-based algorithms for breast cancer classification, utilizing clinically significant features to assist radiologists with numerical representations. Using these features, a graph is generated where the features are represented by the graph nodes, and the relationship among the features is represented by the graph edge. A relationship score is derived using the Pearson correlation technique and a threshold value of 0.2 is selected to determine the node relations based on the model's highest performance. Additionally, the model's performance is evaluated using correlation thresholds of 0.15, 0.2, 0.3, and 0.4. A graph neural network (GNN) model^8^ is proposed to classify breast cancer using the graph data. In a GNN model, each node in the graph is represented by a vector, which encodes information about the node's features or attributes. Similarly, each edge is represented by a vector, which encodes information about the relationship between the connected nodes. Using a GNN model to analyze these features allows us to capture the complex characteristics of breast masses and identify patterns that may be associated with cancer progression. Two GNN models, namely graph attention network (GAT) and graph convolutional network (GCN), are experimented with, using the graph data. The GCN model, which outperforms GAT, is further optimized with an ablation study, fine-tuning the layer structure and hyper-parameters to evaluate the proposed features, a graph, and linear regression equation are presented.

The main contributions of this research can be summarized as follows:

- In clinical practice, perfect assumption of shape, alignment, borders, contiguous tissue structures, and the existence of calcifications shape, and edge features from ultrasound images are necessary and difficult for the radiologist at the same time. Notably, our computerized features can help the radiologist, is time efficient, and can classify the diseases more accurately.
- A graph is generated using these features and using this graph as input a GCN model is proposed and optimized through an ablation study to classify breast tumors. The GCN model can use the structure of the breast features, represented by the nodes and edges of the graph, to capture spatial dependencies.
- With graph-based input, the GCN model outperforms five transfer learning models, a 1D convolutional neural network (CNN) model, ten standalone machine learning (ML) models, and three ensemble-based ML models. It shows superiority over both image-based and feature-based methods. Furthermore, the GCN model is tested on three datasets.
- The correlation among the nodes is evaluated through linear regression. In this method, the y=mx+c formula is utilized to evaluate how one node depends on another for a particular edge, in terms of class prediction.
- In order to improve the reliability and objectivity of our analysis, several variants are utilized, each intended to ensure a more comprehensive assessment of our work. Emphasizing the extraction of clinically relevant features is crucial, as when it comes to medical imaging analysis with deep learning, it becomes crucial to focus on the clinically significant aspects.

## Literature review

Deep learning approaches including CNN and transfer learning have achieved promising results potentially in breast cancer classification tasks using ultrasound images.^9^ BI-RADS is a standardized tool for reporting breast cancer mammograms. Based on BI-RADS, Zhang et al.^9^ implemented a cancer detection system by experimenting with four transfer learning architectures. InceptionV3 gets the highest accuracy of 91.00%. Though deep learning approaches are getting very good accuracy, image pre-processing techniques can be applied for improving performance. With various artifact removal, noise reduction, enhancement procedures, removing undesirable regions can improve the quality Montaha et al.^10^ Customized VGG16 achieves 98.02% accuracy in breast cancer classification.^10^ Attention mechanism in an improved VGG16 architecture combining binary cross-entropy with logarithm of the hyperbolic cosine loss achieves 93.00% accuracy Kalafi et al.^11^ Multilevel transfer learning, feature extraction-based transfer learning , and GLCM-based feature extraction are some of the frameworks which can improve the performance of breast cancer classification (Table 1). Hybrid architectures such as combining VGGNet, ResNet, and DenseNet models were used to develop a robust ensemble architecture for breast cancer classification. Moon et al.^12^ and Tanaka et al.^13^ achieve accuracy of 94.62% and Tanaka et al.^13^ achieves an AUC of 95.01%, sensitivity of 90.9%, and a specificity of 87.0%. These results are further compared to hybrid VGG19 architecture. Another approach is a hybrid architecture combining three pre-trained models to extract deep features. A classification method comes out with these features using feature selection techniques. With this technique Eroglu et al.^14^ achieve 95.6% accuracy. Integrating morphological features with VGG19-based features represents a promising approach to enhance the robustness and accuracy of breast cancer classification systems Daoud et al.^15^ The approach achieves 96.1% accuracy in breast cancer classification. A feature extraction-based technique concat with machine learning (ML) algorithms, a breast cancer classification method designed for low computational complexity Rafid et al.^16^ Notably, the study demonstrated that employing a Random Forest and XGBoost (RF-XGB) classifier stacking method yielded the highest test accuracy among the evaluated models. This finding suggests that the combination of Random Forest and XGBoost, possibly benefiting from the complementary strengths of both algorithms, results in an effective ensemble model for accurate breast cancer classification with 96.03% accuracy. A pre-trained deep learning model, DarkNet-53, is used for extracting intricate and high-level features in the context of breast cancer analysis. DarkNet-53, recognized for its depth and capacity to learn complex patterns, serves as an effective feature extractor. By leveraging the pre-trained weights of DarkNet-53, the model is equipped with the ability to capture hierarchical representations from medical images, enhancing its capability to discern subtle patterns indicative of breast cancer with 98% accuracy Jabeen et al.^17^ GNN model is used to predict the Human Epidermal Growth Factor Receptor 2 (HER2) status of breast cancer based on histopathological images. This innovative approach capitalizes on the power of GNNs to capture intricate relationships and dependencies within the image data, providing a holistic understanding of spatial arrangements and contextual information crucial for accurate HER2 status prediction with 97.05% accuracy Lu et al.^18^ A study integrated a GCN with a relation network (RN) to construct a hybrid model for a specific task, potentially related to breast cancer analysis based on the context provided. This innovative approach combines the strengths of both GCNs and RNs to leverage both graph-based representations and relational reasoning in the learning process. The GCN is adept at capturing complex dependencies and spatial relationships within graph-structured data, making it suitable for scenarios where the input can be represented as a graph, such as in medical imaging Rhee et al.^19^ For breast cancer classification with data security, federated ensemble-based learning approach, combining GNNs with patient-specific profiles, using gene expression data with 88.00% accuracy was used.^20^ This innovative methodology involves creating a graph representation where nodes correspond to specific genes, and edges represent relationships or interactions between them. Importantly, each node is enhanced with patient-specific profiles, providing a personalized touch to the graph structure. Another novel graph representation by leveraging the features of tumors for a specific purpose, potentially related to breast cancer analysis based on the context was provided by Wang et al.^21^ In this approach, each tumor is represented as a graph, where nodes encapsulate distinct features such as tumor shape, orientation, margin, and surrounding tissue characteristics. Research by Yao et al.^22^ presented a novel approach for classifying breast cancer utilizing mammography images. In this method, deep learning techniques were employed, specifically leveraging CNN models to extract deep feature maps from the mammography images. These deep feature maps were then utilized as node features in a graph representation. This model achieves 64.03% accuracy.

**Table 1.** A generalized form of literature review separated by domain of method.

Author	Method	Results obtained	Contribution	Domain of method
Zhang et al. ^9^	Transfer learning method	Accuracy: 91.0%	Classified breast cancer based on BI-RADS, experimenting with four transfer learning architectures. InceptionV3 gets the highest accuracy.	Deep learning models
Montaha et al. ^10^	Classification with VGG16	98.02% with customized VGG16 mode.	A successful technique to classify breast images into four classes. With various artifact removal, noise reduction, and enhancement procedures, undesirable regions were removed, the quality was improved, and the malignant lesions emphasized.	
Kalafi et al.^11^	Attention mechanism in an improved VGG16 architecture	Accuracy: 93%	Attention mechanism in an improved VGG16 architecture and proposed a loss function, combining binary cross-entropy with logarithm of the hyperbolic cosine loss	
(Aswiga, Aishwarya, and Shanthi 2021)^12^	Multilevel transfer learning (MLTL). Feature extraction-based transfer learning (FETL). GLCM-based feature extraction.	ROC curve: 0.89	Proposed two-level framework for breast cancer classification	Handcrafted feature extraction, deep feature extraction
Moon et al. ^13^ Tanaka et al. ^14^	Intigrated several CNN models	Moon et al. ^16^ accuracy= 94.62% and Tanaka et al. ^17^ AUC = 0.951.	Combining VGGNet, ResNet, and DenseNet models to develop a robust ensemble architecture.	
Eroglu et al. ^15^	Hybrid architecture combining three pre-trained models	Accuracy = 95.6%	Implemented a hybrid architecture combining three pre-trained models to extract deep features. Using a feature selection technique, breast cancer was classified with an ML algorithm	
Daoud et al. ^16^	Deep features were incorporated with handcrafted features (texture + morphological).	Accuracy = 96.1%	Combining morphological features with VGG19 based features	
Rafid et al. ^17^	Feature extraction-based technique with machine learning (ML) algorithms	Accuracy = 96.03%	Introduced a feature extraction-based technique with machine learning (ML) algorithms, providing a breast cancer classification method with low computational complexity. They found that a Random Forest and XGB classifier (RF-XGB) stacking method achieved the highest test accuracy	
Jabeen et al. ^18^	Applied a pre-trained model, DarkNet-53, to extract deep features	Accuracy: 98%	Implemented a hybrid architecture combining three pre-trained models to extract deep features. Using a feature selection technique, breast cancer was classified with an ML algorithm	
Lu et al. ^19^	GNN model	Accuracy: 97.05%	GNN model to predict the Human Epidermal growth factor Receptor 2 (HER2) status of breast cancer using histopathological image.	Graph neural network model
Rhee et al. ^20^	Implemented a graph convolution neural network and a relation network to build a hybrid model	Accuracy: 94.70%	The hybrid model was integrated to classify breast cancer using gene expression data.	
Pfeifer et al. ^21^	Federated ensemble-based learning with GNN	Accuracy = 88.0%	Introduced federated ensemble-based learning with GNN to classify breast cancer using gene expression data. The nodes of the graphs were enhanced with patient-specific profiles	
Chen et al. ^22^	Causal graph from the structured data of mammography reports	Accuracy = 73.6%	They integrated a tabular learning technique with causal graphs to preserve the association among features and a GNN was used to combine node information.	
Wang et al. ^23^	a novel graph using the features of tumor	AUC = 96.6%	Designed a novel graph using the features of tumor lesions from pathological images.	
Yao et al. ^24^	GNN model	Accuracy = 64.3%	Proposed a GNN network to classify breast cancer using mammography images. They defined node features using the deep feature maps obtained from a CNN model and an adjacency matrix of the nodes was used to denote the association.	

Prior research mostly focused on classifying breast cancer using deep learning models with raw image input or ML classifiers commonly utilizing image-based features, overlooking the clinically meaningful feature extraction process. These features might not necessarily have a strong relationship with the prognosis of breast cancers identified through ultrasound images. This study only considers those features which are related to clinical diagnosis. Using graph data for the classification can have a great impact as the relation among the features can be presented in a graph and the classification based on the node relations of the graph. In recent studies, limitations remain in representing relevant features into a graph and classifying breast tumors accordingly. Additionally, the studies presented in the literatures did not include any analyses of model performance across multiple datasets for the classification of the same disease. This research presumably attempts to address these limitations significantly to propose an automated and robust breast cancer diagnostic approach.

## Dataset

**Figure 1.**
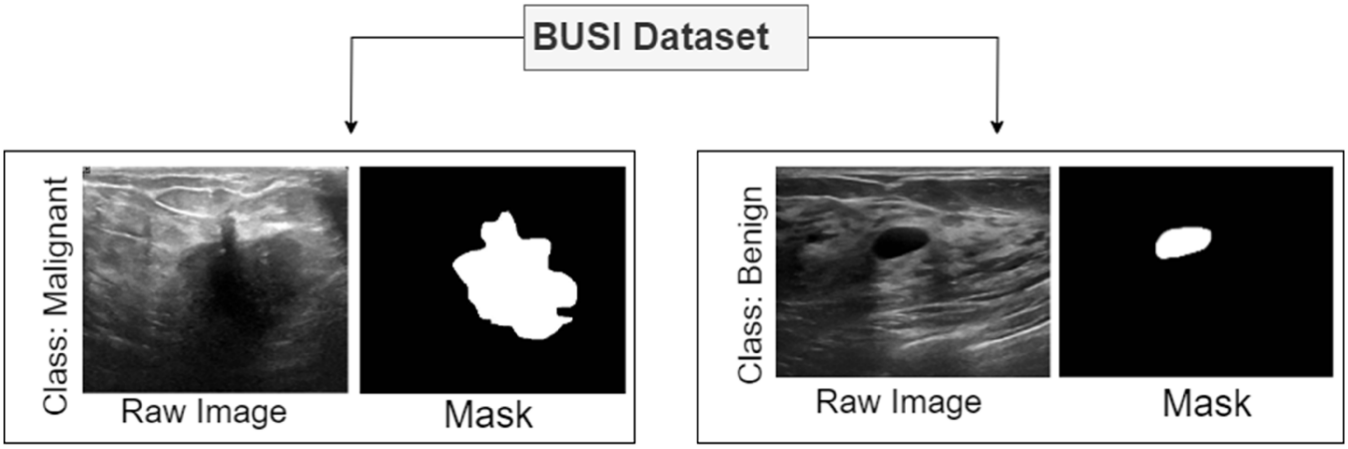
Sample images of the dataset for class benign and malignant.

In this study, a publicly available breast cancer dataset of ultrasound images is used.^25^ This research focuses on Radiology and Imaging in the disciplines of Medicine and Dentistry, particularly dealing with image and mask images. The LOGIQ E9 ultrasound and LOGIQ E9 Agile ultrasound systems provided the data used in this research, guaranteeing a large and thorough dataset for analysis. The PNG format in which the images are stored allows for easy processing and alteration. Within the experimental framework, each image is carefully classified into three groups: normal, benign, and malignant. In the field of radiology, this organized categorization method makes it possible to investigate diagnostic patterns in a more sophisticated manner and makes it easier to create reliable models for better diagnostic and medical imaging services. The dataset contains 780 images of 600 female patients aged between 25 and 75 years, from Baheya Hospital, Cairo, Egypt. The dataset has an average image size of 500×500 pixels, and the images are in PNG format. The images are divided into three classes, normal, benign, and malignant, where for benign and malignant a ground truth is provided. The normal class contains 266 images, the benign class contains 891 images, and the malignant class contains 421 images. Since the objective of this study is to analysis the tumor patterns of breast cancer and in normal cases no tumor is present, only the benign and malignant classes are considered for these experiments. Table 2 shows the dataset details and Figure 1 shows sample images of benign and malignant tumors with their corresponding ground truth.

**Table 2.** The BUSI dataset description.

Class	Image	Resolution	Image format	Patient inclusion range	Patient number
Normal	266	500×500	PNG	25 and 75 years	600
Benign	891				
Malignant	421				

## Methodology

**Figure 2.**
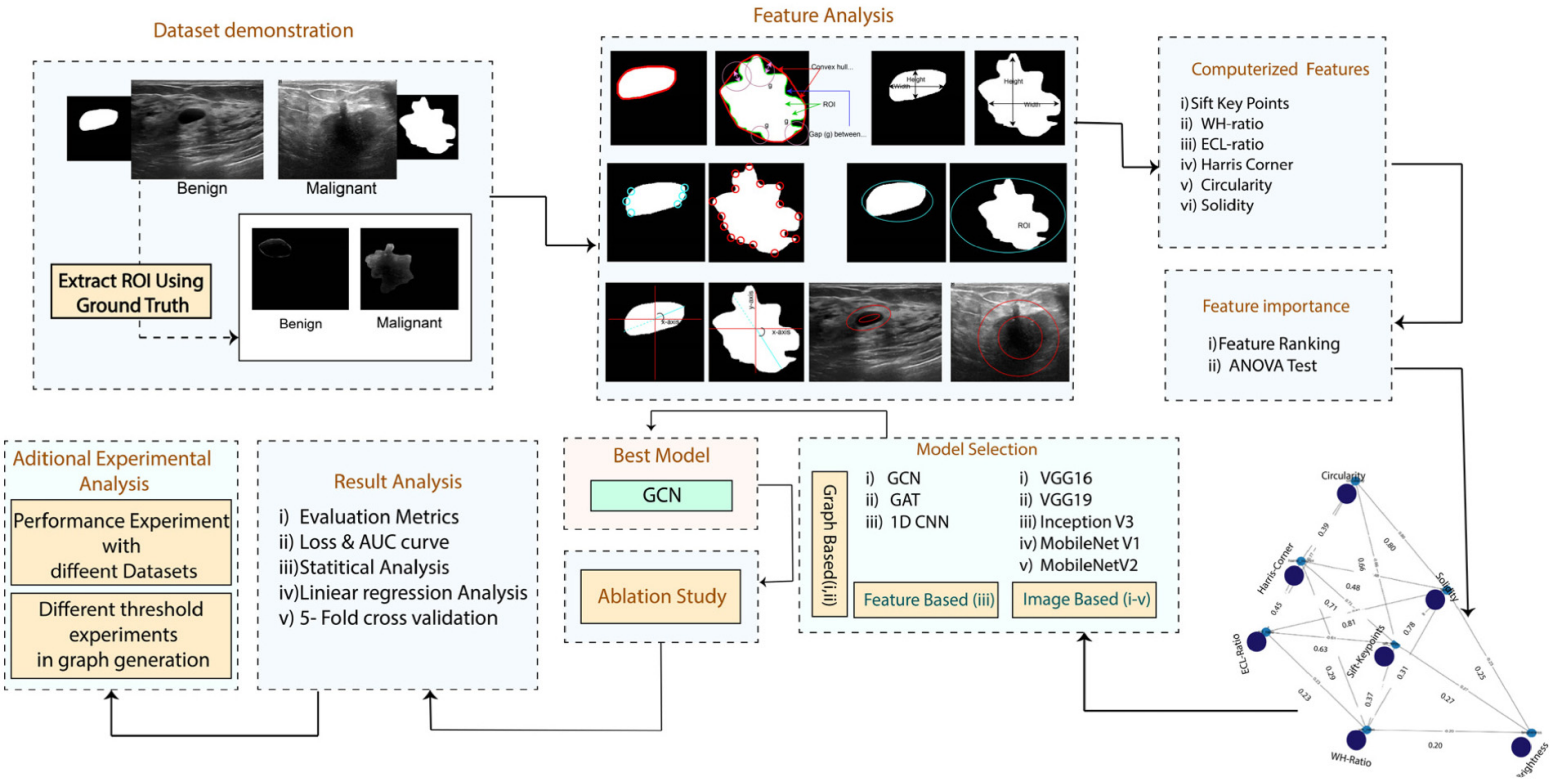
Methodology pipeline of the study.

The key objective of this study is to develop an automated detection system with seven handcrafted clinical markers incorporating an innovative GCN model to aid radiologists and doctors for disease classification. Figure 2 depicts the overall methodology of this study.

Breast ultrasound images of benign and malignant tumors are used, where ground truth masks are provided for each of the classes. The ground truth masks are applied to the original image using a bitwise AND operation to extract the lesions. Seven clinically relevant handcrafted features are extracted from the images: solidity, scale invariant feature transform (SIFT) key points, width-height ratio, elliptic ratio, Harris corner, circularity, and brightness. Several techniques, including ANOVA, Boxplots, and Feature ranking are utilized to assess feature significance. To classify the benign and malignant tumors using a graph-based model, a graph is generated using seven features, applying the Pearson correlation method with a threshold of 0.2. The model's performance is assessed at Pearson correlation thresholds of 0.15, 0.2, 0.3, and 0.4, where a relationship score with a 0.2 threshold is selected. This means that we only include the edges whose node connection attributes have higher weights or are strongly connected to one another. Two graph-based models, GAT and the GCN, are implemented and trained with the graph data (nodes, edges, and edge weights) generated using handcrafted features. Of these two graph-based models, GCN outperforms GAT in terms of classification accuracy. The GCN model is therefore further optimized with ablation study, changing architecture structure and hyper-parameters. The GCN model's performance is compared with image-based classifiers, including five transfer learning models named VGG16, VGG19, InceptionV3, MobileNetV1, and MobileNetV2, as well as feature-based classifiers of a CNN model, ten individual ML models and three ensemble models. Furthermore, the proposed model's performance on three different ultrasound image datasets to classify breast cancer is also evaluated, where two datasets are publicly available and combining them the third dataset is generated. Several performance metrics are applied to analyse the results, the ROC curve is evaluated, and potential overfitting issues are assessed using k-fold cross-validation. The correlation among the nodes is evaluated through linear regression-based equation analysis using the formula, y=mx+c .

## Feature analysis

Both benign and malignant masses have several distinct features. In clinical practice, radiologists examine characteristics related to tumor shape, pattern, and color intensity to determine the cancer stage.^26^ Collectively, these features help to provide an in-depth study of breast ultrasound images by obtaining numerical representation of data about structure, geometry, and intensity. These features’ capacity to draw attention to unique patterns linked to abnormal growth, asymmetric shapes, and other indicators of malignancies is what relates them to the classification of benign and malignant classes. According to previous studies, clinical biomarkers can differentiate between benign and malignant instances with greater accuracy and robustness when it considers various aspects of the images.^1^ Although, this study attempts to analyze breast masses by deriving computerized features based on clinical markers. Table 3 describes the computerized features which are extracted following the clinical biomarkers.

**Table 3.** Clinical biomarkers and computerized features of breast tumors.

No.	Clinical markers	Computerized features	Description
1	Curviness of tumor edge	Convex hull ratio/solidity	a measure of the compactness of an object in the image
2		Scale invariant feature transform (SIFT) extreme points	a descriptor that characterizes an image region based on its local gradient orientation histograms
3		Harris corner points	a measure of the image's local structure that is useful for feature detection
4	Shape of the tumor	Elliptic ratio	the ratio of the eccentricity of an object to its compactness
5		Circularity	a measure of how closely an object resembles a perfect circle
6	Lateral dimension	Width-height ratio	the ratio of the width of an object to its height
7	Transition of tumor margin	Brightness	the average intensity of the image pixels in a certain region

**Figure 3.**
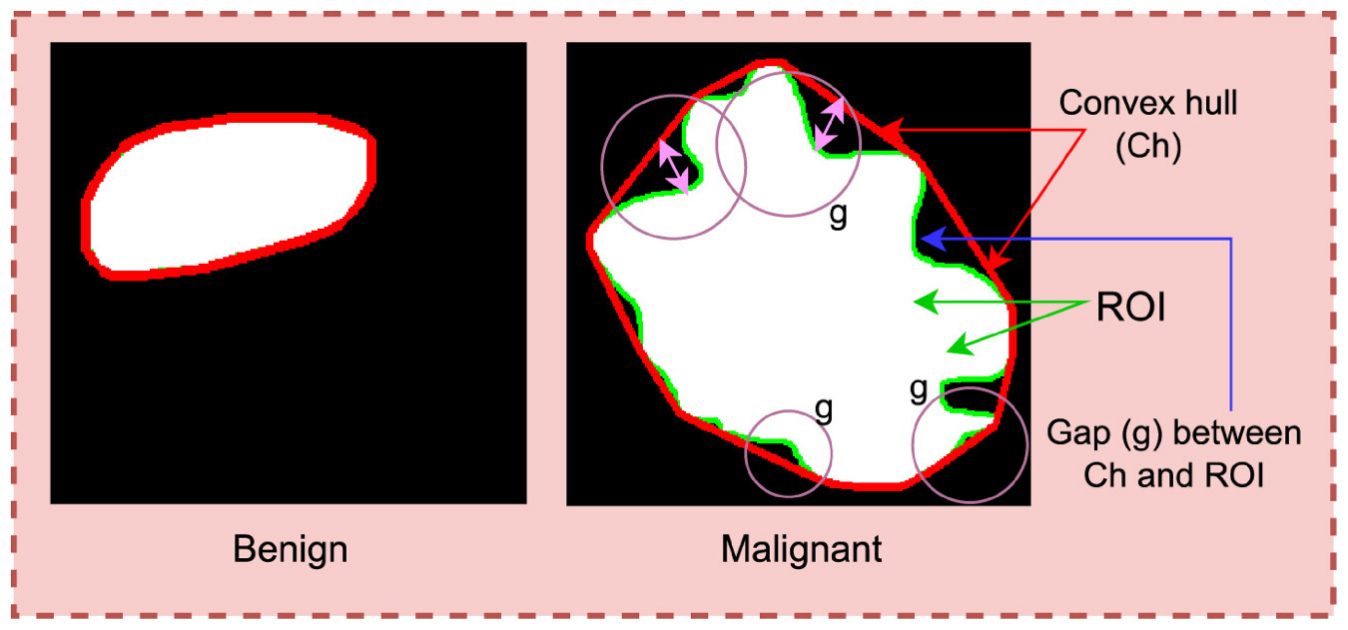
Solidity of the tumor comparing the convex hull and tumor edge.

**Figure 4.**
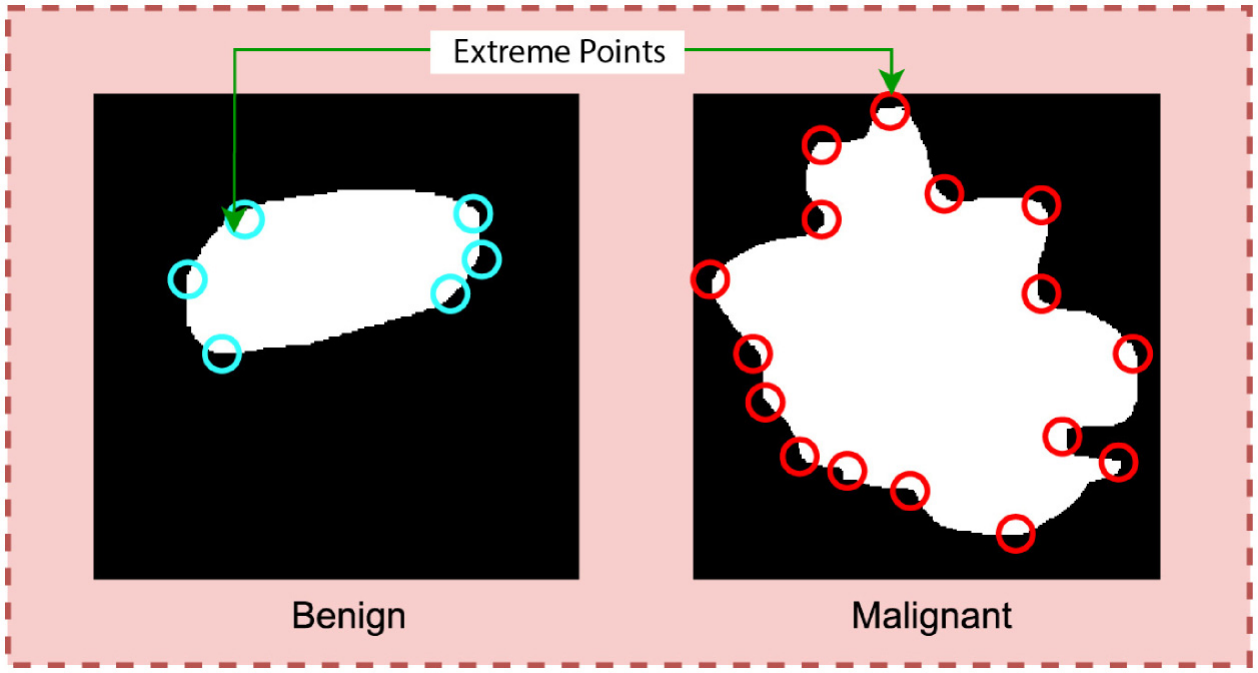
SIFT extreme points extraction from the tumor.

**Figure 5.**
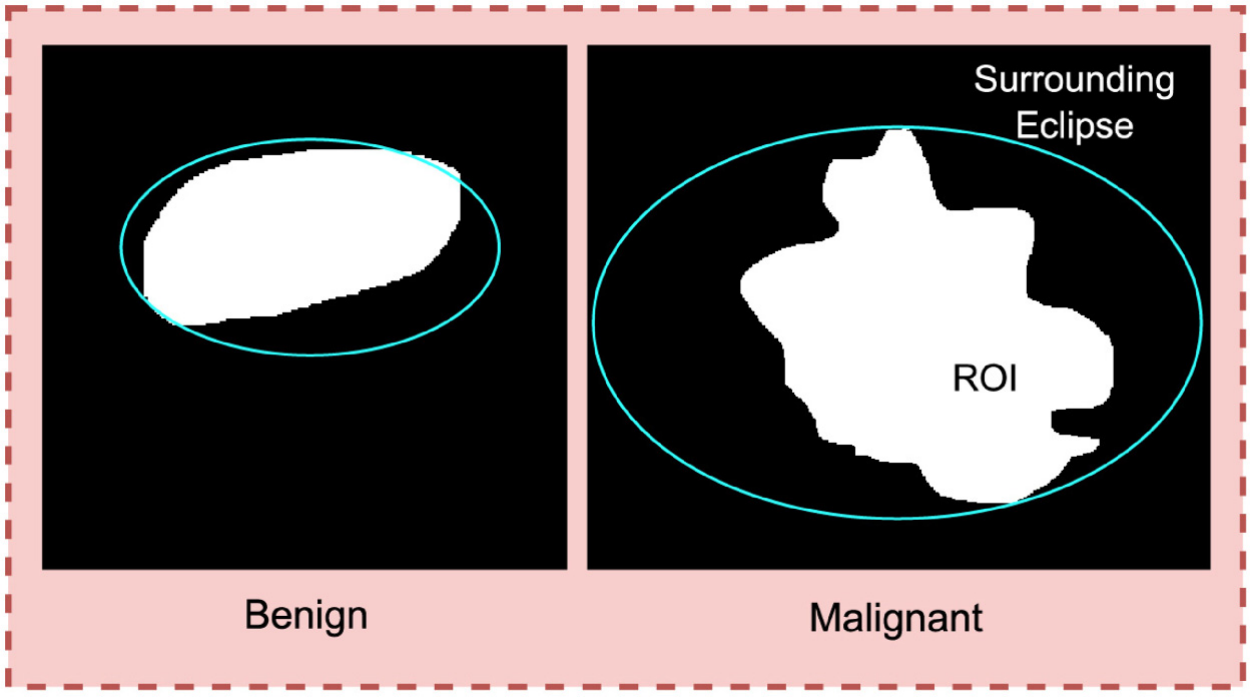
Deriving eclipse ratio of the tumor.

**Figure 6.**
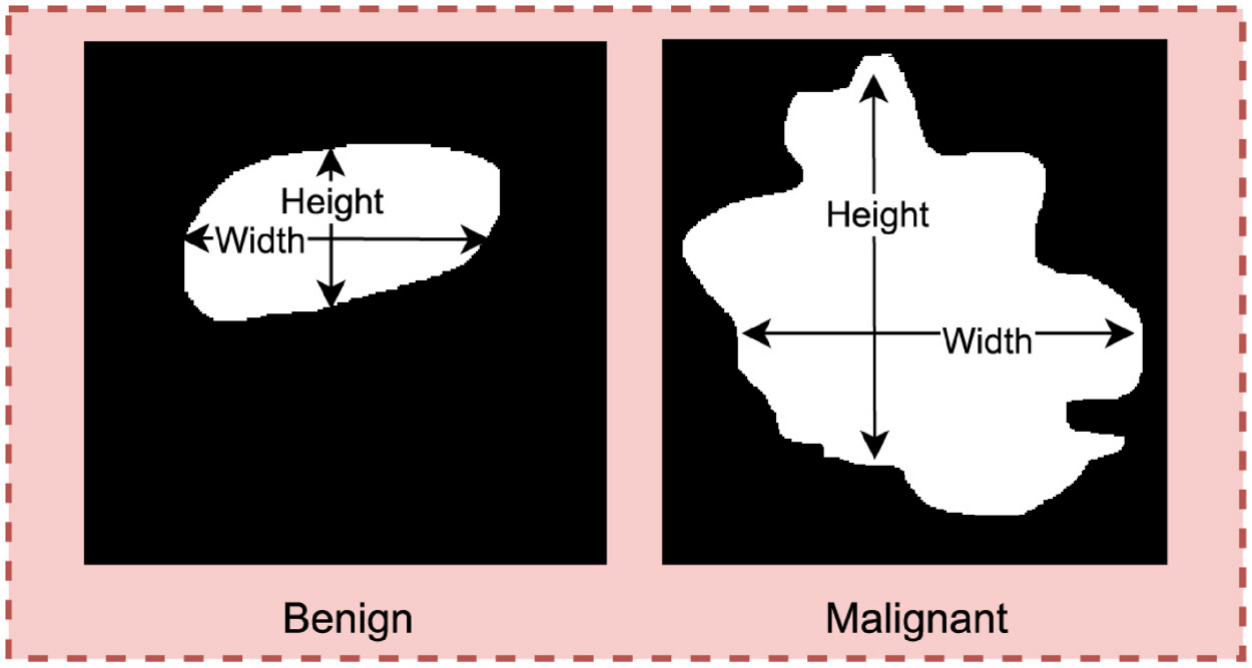
Deriving width/height ratio of the tumor.

**Figure 7.**
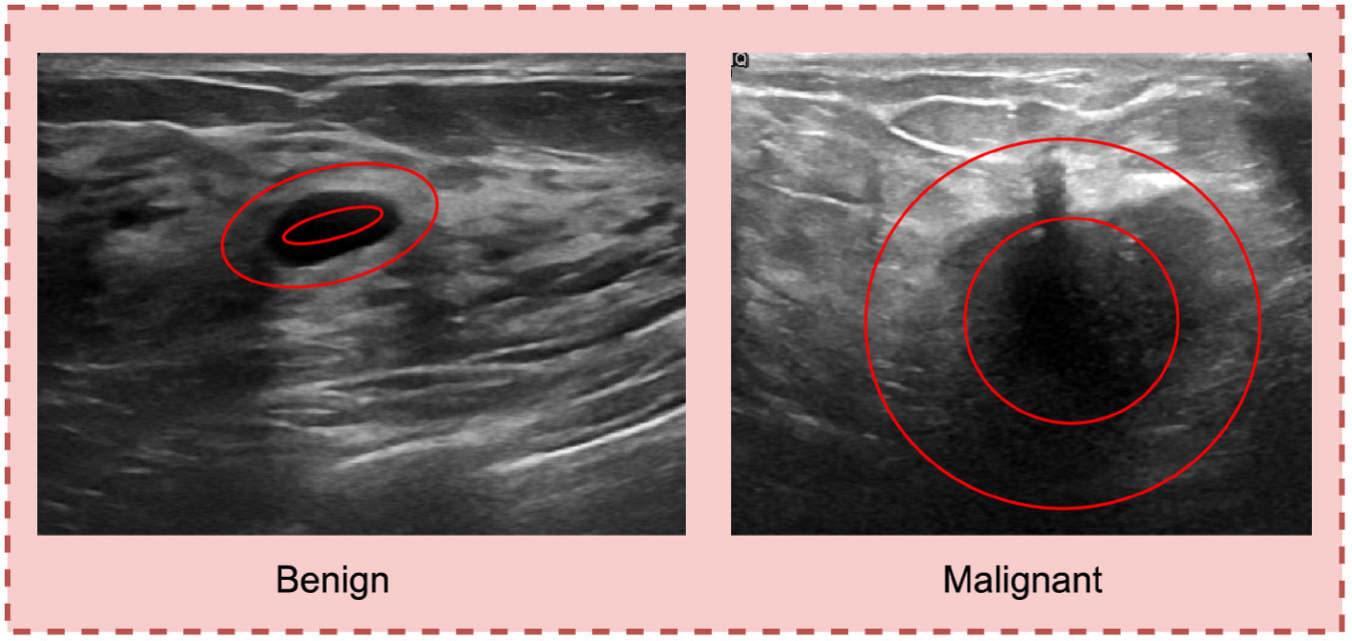
Transition of tumor margin evaluation through deriving pixel brightness.

It can be observed from Table 3 that to determine breast cancer, several tumor characteristics are assessed clinically. These characteristics include curviness of the tumor edge, shape of the tumor, pattern of lateral dimensions, parallelism of the tumor and transition of the tumor margin. To evaluate these clinical markers with computerized features, seven handcrafted features are extracted from the tumorous region of the images. The computerized features include convex hull ratio, SIFT extreme points, Harris corner points, elliptic ratio, circularity, width-height ratio, and brightness. The curviness of tumor edge is represented by solidity,^27^ SIFT extreme points, and Harris corner points. The shape of tumor is represented by elliptic ratio and circularity. The lateral dimension is represented by width-height ratio and transition of tumor margin is represented by pixel brightness. In summary, Figures 3 to 7 illustrate feature extraction from the images.

### Curviness of the tumor edge

Analyzing the Region of Interest (ROI) of both benign and malignant masses, it is observed notably that the margin of a benign mass is less curvy than the margin of a malignant mass.^28^ The benign tumor has a smooth and well-defined border whereas the malignant tumor presents a curvy pattern. To analyze the curviness, three features, (i) convex hull ratio, (ii) SIFT extreme points, and (iii) Harris corner points are extracted. Figure 3 illustrates the methods of representing the solidity of tumor.

In Figure 3, the red and green borders outline the convex hull and ROI. The gap between these borders indicates tumor curvature. A wider gap indicates greater curvature, noticeable in malignant tumors, while benign ones show such a minimal gap, that the hull and ROI border overlaps. The value of the feature is computed following equation 1.

(1) Convexhullratio(CHR)=CH/TA

where, CH is the convex hull of the object and TA represents the tumor area enclosed by the active contour. The values for both of parameters are calculated using equation (2).

(2) $$A=\frac{1}{2}\overset{n-1}{\underset{i=1}{\sum }}\;(x_{i+1}-x_{i})(y_{i+1}-y_{i})$$

Here, the expression $x_{i+1}-x_{i}$ represents the difference between the value of *x* at index i+1 and the value of *x* at index *i*. Similarly, $\left(y_{i+1}-y_{i}\right)$ represents the difference between the value of *y* at index i+1 and the value of *y* at index *i*. The product of these two differences represents the area of a rectangle. The summation symbol ∑ indicates that we need to sum up these signed areas for all values of *i* from 1 to n −1.

Calculating extreme points can be another effective approach to find the curviness of an object. Extreme points refer to the topmost, bottommost, rightmost, and leftmost points of an object. Figure 4 is a visualization of the extreme points of an object.

Typically, the number of extreme points will be greater for a curvy object than for a less curvy object. The degree of curviness can be measured by extracting extreme points from the benign and malignant ROIs integrating two algorithms: SIFT extreme points and Harris corner point detection.

The SIFT algorithm identifies the local features of an image,^29^ also termed “key points.” The method transforms the data of an image into scale-invariant coordinates. In this process, points of low and uneven contrast are eliminated. Equation (3) is used to locate extreme points using the SIFT algorithm.

(3) $$Z=-\frac{\partial^{2}D^{-1}}{\partial x^{2}}\;\;\frac{\partial D}{\partial x}$$

This equation involves partial derivatives and includes the second derivative of quantity *D* with respect to *x*, denoted as $\left(\frac{\partial^{2}D^{-1}}{\partial x^{2}}\right)$ , multiplied by the partial derivative of *D* with respect to x , denoted as $\frac{\partial D}{\partial x}$ , and negated (multiplied by $D^{-1}$ ). In summary, this equation represents a product of the second derivative of *D* with respect to *x* and the partial derivative of D with respect to x . The negative sign indicates the direction of change. The Harris Corner detection algorithm^30^ was designed to detect the inner corners of object which generally describes the patterns of the border, including curviness. In this algorithm, the corners are identified based on the greatest intensity variations across all magnitudes and directions.

### Shape of tumor

Benign masses tend to be round or ellipsoidal.^31^ In Figure 5, benign tumors appear as smooth and ellipsoidal, while malignant tumors have irregular shapes. To distinguish the two types of tumors based on shape and the circularity, eclipse ratio are derived.

Circularity is a measure which is used to assess the similarity of the tumor shape to that of the circle.^32,33^ The feature can be calculated using equation (4).

(4) $$F_{circ}=1-\left(4\pi \;\frac{A}{P^{2}}\right)$$

where, *P* refers to the perimeter of the closed contour and *A* is the contour area, calculated according to equation 5. The contour area is found by counting the pixels number inside the tumor region. The perimeter can be measured by counting the number of pixels of the tumor boundary.

(5) $$P=\overset{n-2}{\underset{i=1}{\sum }}\;\sqrt{\left(x_{i+1}-x_{i})+(y_{i+1}-y_{i}\right)}$$

To derive the eclipse ratio, based on the coordinates of the tumor edge, an elliptical curve is drawn around the tumor, as shown in Figure 5 (cyan line).

The elliptical curve covers the entire tumor area. Therefore, the gap between the tumor edge and the ellipse will be greater for non-elliptical objects and smaller for shapes closer to an ellipse. The eclipse ratio can be calculated as shown in equation (6).

(6) $$Eclipse\;ratio(ER)=\frac{ECA}{TA}$$

where ECA represents the area within the elliptic curve and TA represents the tumor area, enclosed by the active contour.

### Lateral dimension

Benign tumors are often wider than taller. In other words, the lateral dimension of benign mass is larger than anterior-posterior dimension.^26^ As can be seen in Figure 6, the width of a benign tumor tends to be greater than its height whereas the malignant masses are found to be taller than wider.

To evaluate the lateral dimension, the width/height ratio of the tumor is derived, using equation 7.

(7) $$Width-height\;ratio(WHR)=\frac{W}{H}$$

where, *W* denotes the width of the tumor contour and *H* represents the height of the tumor.

### Transition of tumor margin

To distinguish benign and malignant tumors, the margin of the tumor is considered a significant marker. In the case of benign masses, a thin, smooth, and clearly defined^28^ margin is observed. A sharp transition between the tumor and the surrounding natural tissue is visible, as can be seen from Figure 7.

In contrast, for the malignant lesion, no clear transition between the tumor and neighborhood cells is found and the border is less defined. To extract features based on transition of the tumor margin, the surrounding area of tumor is first segmented. From the border of the tumor, 10 pixels of the inner area and 10 pixels of the outer area (altogether 20 pixels) of the tumor are considered. Brightness, which is a photometric feature, is extracted from the segmented region.

## Feature importance

Feature importance and pattern analysis are two important tasks in ML and data analysis.^34^ Identifying key features may improve model performance and enhance understanding of complex data like medical images with better predictions and decisions. Since seven extracted features are considered as computerized clinical features, the significance of features in terms of classifying benign and malignant cases is analyzed with ANOVA and feature ranking to assess the importance of the features.

### ANOVA test

**Figure 8.**
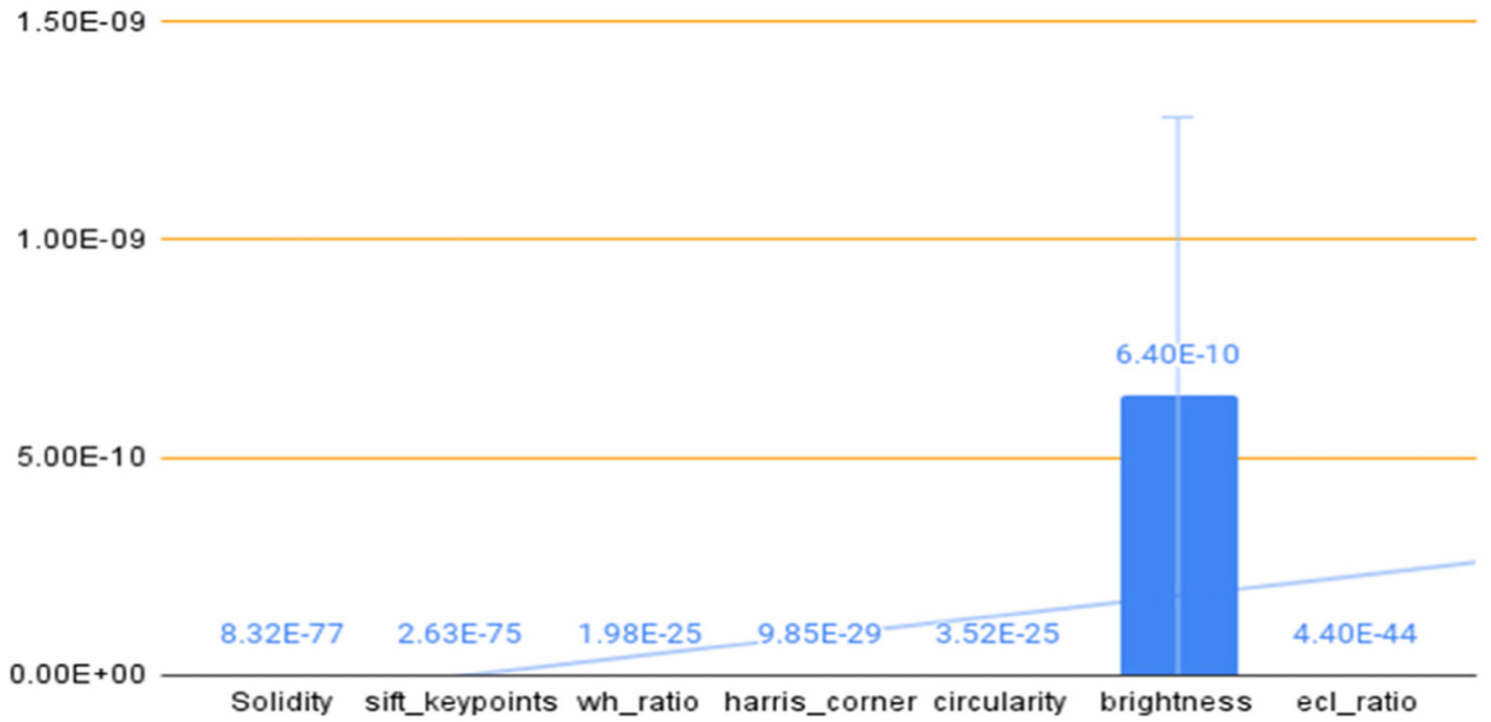
Visualization of ANOVA test results.

ANOVA is a statistical test that measures the significance of differences between groups of data. By applying ANOVA to the features, we can identify which features are most significant in explaining the differences between different groups or classes in our dataset. This can be particularly useful for identifying which features are most informative for classification or regression tasks.^35^ The outcome of ANOVA tests stated that the proposed features are highly significant in terms of distinguishing the classes. The alternative hypothesis in an ANOVA is that at least one of the group's means is different; while the null hypothesis is that the means of all the groups are equal. The *p*-value from an ANOVA indicates the probability of obtaining the observed result if there is no real difference between the groups being compared. If the *p*-value is lower than the significance level of 0.05 in feature selection, we reject the null hypothesis. This suggests that the means of the groups are not equal, and therefore the feature is significant. Figure 8 describes the visualization of the results.

All of the features, however, are significantly less than 0.05. This shows that all features reject the null hypothesis and are sufficiently significant when compared to other features.

### Feature ranking

**Figure 9.**
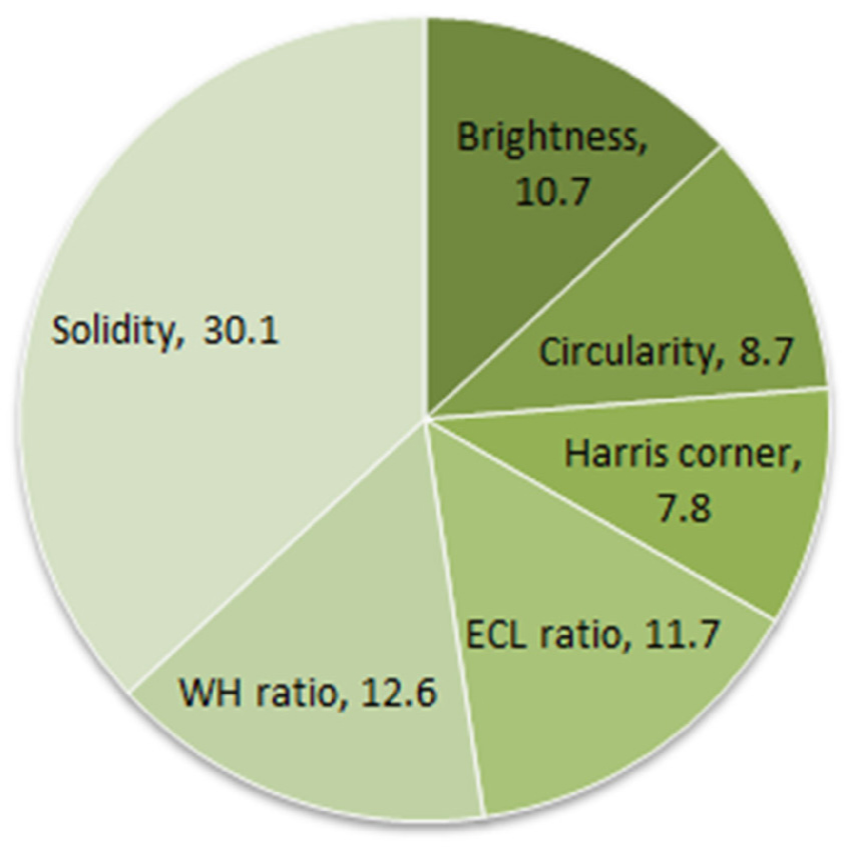
Feature impact analysis based on feature ranking using SVM.

Feature ranking using linear support vector machine (SVM) is a commonly used method in ML for identifying the most important features in a dataset that contribute to the classification or regression task. It works by fitting a linear SVM model to the data, then using the coefficients of the SVM model^36^ to rank the importance of each feature. Figure 9 illustrates the bar chart of feature ranking using SVM.

It can be observed from Figure 9 that all the features have a ranking in a range of 8–30% which validates the significance of all features. After experimenting with various methods for feature selection and pattern analysis, we have found that the derived features are highly informative and have strong discriminative power. By including these features, an improved classification performance can be expected from the model.

## Generate a graph with features

Our GNN model is based on seven handcrafted features extracted to capture important characteristics of breast ultrasound images. The aim is to create a graph with these features. According to graph definition, a graph is represented as: G=(V,E) where *V* is the set of vertices or nodes (we will use the handcrafted features as nodes) and *E* is the set of edges. Here, $V_{i}\in V$ to denote a node and $e_{ij}=(V_{i},V_{j})\in E$ to denote an edge pointing from $V_{i},V_{j}$ . The neighborhood of a node *V* is defined as N(V)={u∈|(v,u)∈E} . The adjacency matrix *A* is a n*n matrix with $A_{ij}=1$ if $e_{ij}\in E\;$ and $A_{ij}=0$ if $e_{ij}\in E\;$ .^11^ For the graph generation, the seven handcrafted features are utilized. Each feature is represented as a node and the set of the nodes is represented as {V} in the graph. The connection of each node represents the set of edges {E} as per the graph definition. The nodes are connected with the edges based on their similarity, connections, and co-relations. Nodes that are similar are more likely to be connected. Pearson correlation is a statistical measure that quantifies the degree of linear relationship between two variables.^37^ It is commonly used in data analysis to assess the strength and direction of association between variables. In our proposed GNN model, the Pearson correlation method is applied to measure the similarity between nodes in the graph. By calculating the Pearson correlation coefficient between pairs of nodes, the degree of the connection between them is determined. To calculate the Pearson correlation coefficient, the values of each feature are standardized first by subtracting the mean and dividing by the standard deviation. The covariance between the two standardized variables is then calculated and divided by the product of their standard deviations. Pearson correlation is calculated according to equation 8.

(8) $$r=\frac{\sum (x_{i}-\;x^{\prime })(y_{i}-y^{\prime })}{\sqrt{\sum {\left(x_{i}-x^{\prime }\right)}^{2}{\left(y_{i}-y^{\prime }\right)}^{2}}}$$

**Figure 10.**
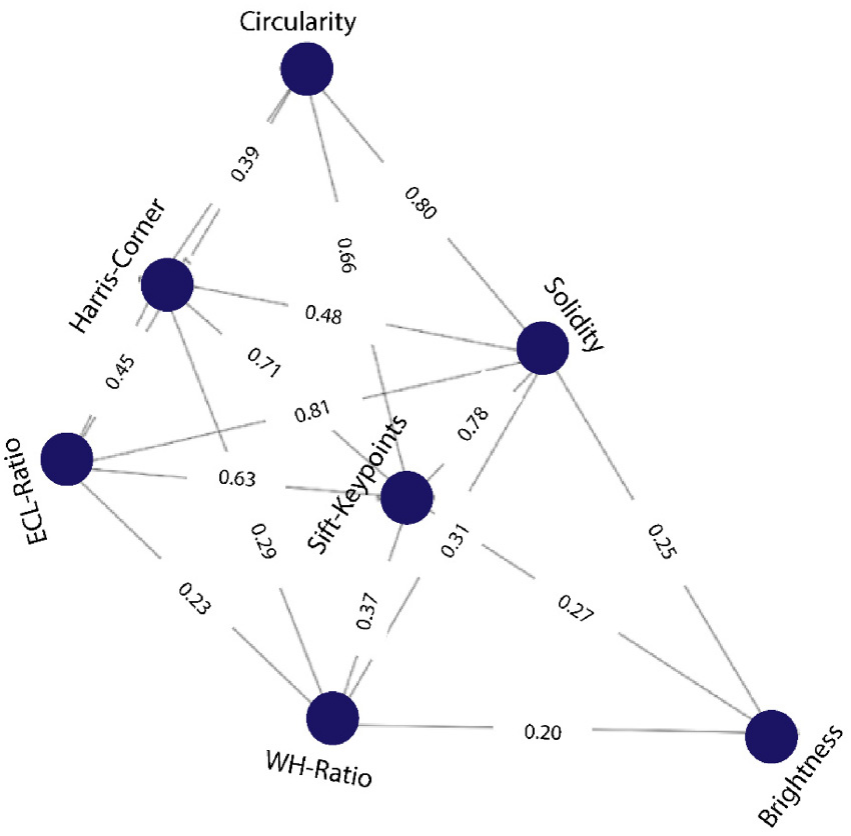
Representation of the graphs with nodes, edges, and edge weights.

The resulting values range from −1 to 1, with a value of −1 indicating a perfect negative correlation, 0 indicating no correlation, and a value of 1 indicating a perfect positive correlation. In our GNN model, a high Pearson correlation coefficient between two nodes indicates a strong connection, while a low coefficient indicates a weak connection. To determine edges between nodes in the graph, a threshold value of 0.2 is used for the Pearson correlation coefficient. Through experiments, a suitable threshold of 0.2 has been found to balance feature retention with accuracy. The accuracy slightly drops when the threshold is set to > 0.2, indicating that adding more edges or features will increase noise in the final graph. When the threshold is set to 0.2 or higher leads in the disconnections of several important features from the graph such as WH-ratio, solidity. The selection of 0.2 is considered as the optimum since it reduces these trade-offs and offers a threshold value that best keeps accuracy while keeping essential features. An algorithm is shown in Algorithm 1 by which the edges are selected with the threshold value and the experiment of selecting the threshold value is explained in the result section. If the coefficient between two nodes exceeds this threshold, an edge is added between them. This creates a graph where highly correlated nodes are more likely to be connected. Incorporating this information into the GNN model enhances prediction accuracy by effectively capturing the underlying data structure. Tabular data derived from the graph represents the nodes, edges, and edge weights (Pearson correlation values). This tabular data generation is an important element of using a GNN model, as it allows feeding the graph structure into the model and making predictions based on the connections between nodes. In Figure 10, the graph is represented with nodes, edges, and edge weights and in Table 4 an example of the tabular data for the input of the GNN model is shown.

**Table 4.** Tabular data which is generated from the graph for the input of the GNN model.

Features	Nodes	Edge (source)	Edge (target)	Edge weight
Solidity	1	1	2	0.78
SIFT key points	2	1	3	0.31
Width-height ratio	3	1	4	0.81
Ecliptic ratio	4	1	5	0.47
Harris corner	5	1	6	0.79
Circularity	6	1	7	0.25
Brightness	7	2	3	0.36
		2	4	0.62
		2	5	0.7
		2	6	0.64
		2	7	0.27
		3	4	0.22
		3	5	0.29
		3	6	0.21
		3	7	0.43
		4	6	0.76
		5	6	0.39

Algorithm 1: Creating the Edges for the graph with optimal the threshold value

***Begin***
 *Nodes TO Get Column Names (DataFrame)*
*  Edges TO []*
*  FOR i IN range (0, len(DataFrame.columns))*:
*    FOR j IN range (i + 1, len(DataFrame.columns))*:
*      IF i <* j
*        Correlation = DataFrame[col1]. corr(DataFrame[col2])*
*        IF abs(corr) > TO 0.2:*
     *Edges. Append*((column_name[i], column_name[j]))
 *        ENDFOR*
*  *ENDFOR**
*  G TO Create Empty Graph ()*
*  Add Nodes to Graph (G, Nodes*)
*  Add Nodes to Graph (G, Edges)*
*  Pos TO Get Graph Layout(G)*
*  fig, a×TO Create plot ()*
*  Draw Network (G, pos, ax)*
*  Edge labels TO calculate edge labels (G, DataFrame)*
*  Draw edge labels (G, pos, edge labels, ax)*
    Hide axis(ax)
***END***

The tabular data consists of five columns: Features, Nodes, Edge (source), Edge (Target), and Edge Weight. The Edge (source) and Edge (Target) columns represent the nodes in the graph that are connected by the edge, and the Edge Weight column represents the strength of the connection between them. These graph data are used as a tuple as input of the GNN model. For example, in the first row, the first feature, solidity, is shown as node 1. Node 1 (solidity) is connected to node 2 (SIFT key points) with a Pearson correlation score of 0.78. Hence, in the third column the edge source is node 1, in the fourth column the edge target is node 2, and in the fifth column the edge weight is 0.78.

## Classification

As stated previously, the classification of breast tumors is carried out using a graph-based GCN model. The performance of the proposed graph-based model is further compared with another GNN model named GAT, state of art ML, ensemble ML and DL models including a 1D CNN and five transfer learning models. Therefore, the GAT and GCN models are trained on graph data, ML and ensemble ML are tested on hand-crafted features, and DL models are experimented with ROI images of benign and malignant cases.

### GCN model

GCN is a general framework, a subclass of deep neural networks, designed with inference on graph-based data. The graph data is represented by nodes, edges, and edge weights which are the input of the model. The main objective of GCN architecture is to learn embedding neighborhood information.^38^

#### Feed-forward network layer

**Figure 11.**
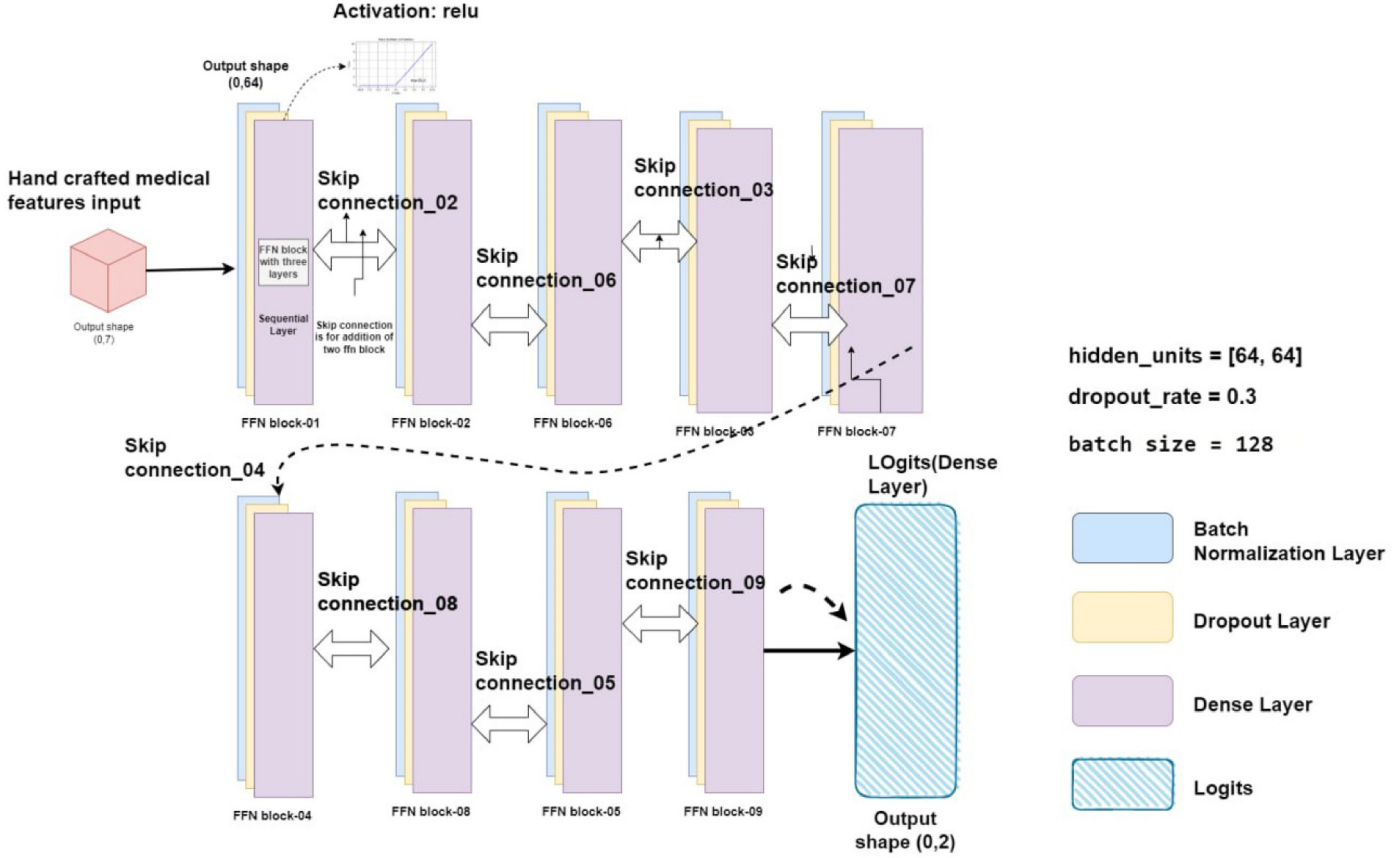
Representation of the FFN layer with optimized FFN layers and nine skip connections.

Specifically, to improve the efficiency of node representation learning, an improved feed-forward neural network (FFN) is integrated in the proposed GCN architecture. This FFN is a predefined classifier with tightly connected layers, as explained by Gayathri et al. in 2022. This architecture works well for identifying complex connections and patterns in the data. Batch normalization layer, dropout layers, and dense layer make up an efficient FFN, which is a kind of one-directional network. Starting from the batch normalization layer, the information flows through multiple hidden levels of change presented as dropout layers. Each layer applies a distinct set of weights and biases to the dense layer.^39^ An entire FFN block is composed of these batch normalization layers, dropout layers, and dense layers. In a GCN model, the use of optimized FFN blocks is essential for learning complex non-linear changes of node representations. Consequently, this improves the model's ability to identify complex patterns in the graph data. The FFN layer connections with FFN blocks architecture is shown in Figure 11.

The architecture of the optimized FFN layer for this study is shown in Figure 11. The FFN is made up of batch normalization layer that receives the input features and passes them through nine FFN blocks, each of which has three fully linked layers and nine skip connections. There are nine FFN blocks in the proposed GCN model, and each block has corresponding skip connections. Information can move straight from one block to another without changing hands thanks to a skip connection. The first layer is a batch normalization layer, which normalizes the input data to improve model performance and prevent overfitting. The second layer is a dropout layer with a dropout rate of 0.3. This layer randomly drops out a percentage of the nodes during training to prevent overfitting and improve model generalization. The third layer is a dense layer with the activation function Gaussian error linear units (GELU). The GELU activation function is known for its ability to improve model performance by mitigating the vanishing gradient problem, which is common in deep neural networks. This helps to ensure that the model can efficiently process large amounts of data and achieve high accuracy.

#### Baseline GCN architecture

The graph of this study can be considered as a multi-relational graph since we have represented different types of features as nodes and these nodes are connected to one another. A multi-relational graph is represented with G=(V,E) where *V* represents nodes and *E* represents the edges. In our model, we consider a tuple for graph data as graph info = (node features, edges, edge weights). The input of the graph G=(V,E) is graph info = (node features, edges, edge weights).^10^ The nodes need to pass the information; hence a message passing framework is used where a single node aggregates messages from its local neighborhood. The messages coming from the neighbors are based on information aggregated from their respective neighborhoods. Based on the surrounding details, each node is assigned to an individual embedding. The GCN layer's convolutional operation is used for message passing. The following equations represent the message transmission procedure.

(10) $$x_{u}^{i}=\sigma \;\left(\;W_{n}^{i}\;x_{u}^{i-1}+W_{Neibour}^{i}\;\underset{v\epsilon N(u)}{\sum }\;x_{v}^{i-1}+b^{i}\right)$$

(11) $$x_{u}^{\left(i+1\right)}=Update^{\left(i\right)}\;\left(x_{u}^{\left(i\right)},\;AGGREGATE\;\left(i\right)\left(\left\{x_{u}^{\left(i\right)},\forall_{u}\in N(u)\right\}\right)\right)$$

(12) $$=Update^{i}\;\;(x_{u}^{i}\;,\;m_{N(u)}^{\left(i\right)})$$

In the equational principals of message passing procedure $x_{u}^{i}$ represented as $i^{th}$ nodes hidden embedding. The node embedding it had before was $x_{u}^{i-1}$ . $W_{n}$ , $W_{Neibour}$ , and *b* represent the parameters passed to the message at iteration *i* on the other hand, elementwise non-linearity is shown by σ . $\underset{v\epsilon N(i)}{\sum }\;x_{v}^{i-1}$ is the next-door node of the embedding vector of *u* ( N(u)) . Equations (11) and (12) explain how the node embedding or message transmission is updated. $m_{N(u)}^{\left(i\right)}$ is the message that aggregated from the neighbor, *N*(*u*). There will be an upgrade to the previous hidden embedding from $h_{u}^{\left(i\right)}$ to $h_{u}^{\left(i+1\right)}$ (updated embedding). To obtain the most important node feature vector, the updated node embeddings are normalized. The final non-linear node embeddings are fed into the densely linked FFN layer after GCN processing. The inputs for the last prediction task are subsequently evaluated for non-linear adjustments by the FFN layer. An Exponential Linear Unit (ELU) activation function is used by the dense layer hidden units, enabling the model to learn complex, nonlinear correlations between the input features. The GCN model's output can be shown as follows.^10^

(15) $$R=F(x_{u},\;f_{u})$$

**Figure 12.**
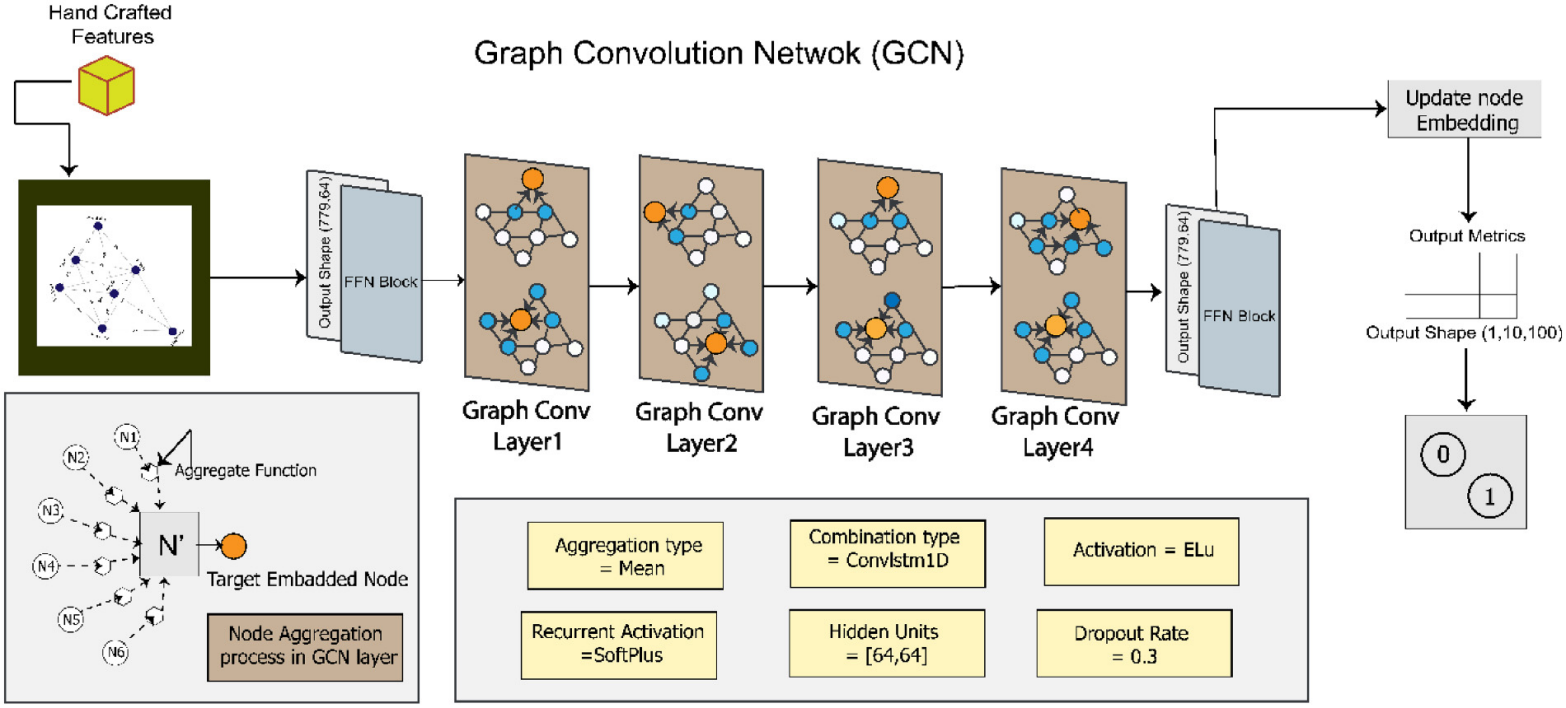
Proposed GCN architecture after ablation study.

In equation (15), *R* is the FFN's predicted label. The function $\left\{F(x_{u},\;f_{u})\right\}$ depicts the feed-forward network in which for every node *u*, the corresponding node embeddings and feature vectors are denoted by $x_{u}\;and\;f_{u}$ . Figure 12 shows the proposed GCN model framework.

In the baseline model, two GCN layers are activated by the SoftMax function. The output of the GCN layer is then passed to the FFN block, which predicts the result in the dense layer. From the dense layer, a metric is generated by the node embedding information. Using this metric, the outcome of the model can be determined. The hidden unit size is [32,32]. The combination function is ConvLSTM1D, and the rectified linear unit (ReLU) is used as the activation function. Similar to the GAT model, the baseline GCN model is trained for 200 epochs with optimizer Adam, learning rate 0.001, and batch size 64.

#### Proposed GCN architecture

The proposed GCN model's performance is enhanced by fine-tuning the baseline GCN architecture through an ablation study. These components include hidden units, the learning rate, the batch size, the dropout rate, the number of GCN layers, the activation function, and the combination function. Figure 12 illustrates our proposed GCN model, with the changes made to the baseline architecture to get the optimized configuration.

By experimenting with different values for each component, the configuration that yields the highest accuracy is determined. The hidden units are set to [64, 64], which means there are two hidden layers in the model, each containing 64 units. This configuration allows interpreting complex representations of the data and improves the model's ability to capture intricate patterns. The learning rate is changed to 0.01, the batch size to 128 with a dropout rate of 0.3 and optimizer Nadam. Four GCN layers are incorporated, enabling the model to capture complex relationships and dependencies within the graph structure. ELU is applied as the activation function. Finally, ConvLSTM1D is added which allows the model to effectively process temporal information in sequential data.

### Comparison with other models

#### GAT

There have been several attempts to adapt neural networks to deal with arbitrarily structured graphs. Many sequence-based activities nowadays make use of attention processes.^5^ The advantage of attention mechanisms is to handle inputs of different sizes while concentrating on the most important information for decision making. Self-attention or intra-attention describe an attention process that computes a representation of a single sequence.^41^

The GAT model is a combination of the two sequential dense layers and two sequential multi-head graph attention layers. The dense layer activation function is “ELU” which is connected with 128 hidden units and 8 heads (128*8). The multi-head graph attention layer is also connected with 128 hidden units and 8 heads. The outcome of the network is a metric with node indices. The model is trained for 200 epochs with optimizer Adam, learning rate 0.001, and batch size 64.

#### 1D CNN and transfer learning models

A 1D CNN model is a neural network that is used for evaluating sequential data. A 1D CNN model is incorporated in this research to compare the performance with the proposed GCN model. In addition, five transfer learning models, named VGG16, VGG19, InceptionV3, MobileNetV1, and MobileNetV2, are trained with the image dataset for comparison. The proposed CNN model is composed of seven layers: four convolutional layers, two Maxpool layers, and one dense layer. The input layer is embedded with the first convolutional layer. The convolutional layer uses a kernel size of 2×2, a filter size of 2×2, the ReLU activation function, and a Maxpool layer with a size of 2×2. Maxpool layers are added immediately after the second and fourth convolution layers. The dense layer is equipped with the sigmoid function. The CNN and the transfer learning models are trained for 200 epochs with optimizer Nadam, learning rate 0.01, and batch size 128.

#### ML and ensemble ML models

The proposed GCN model is further compared with ML and ensemble ML models. The random forest classifier (RFC) uses a group of decision trees to perform classification tasks. Extra trees classifier (ETC) is an unpredictable forest variation renowned for its unpredictable feature selection. Gradient boosting classifier (GBC) develops weak learners one after the other to produce a powerful prediction model. The bagging classifier (BC) combines the predictions of several base estimators. SVMs use hyperspaces to divide data points into groups for classification purposes in high-dimensional space. For classification tasks, SVC is a particular implementation of SVM. K-nearest neighbors (KNN) is a data point classification algorithm that uses the majority class among its closest neighbors. Decision tree classifiers (DTCs) base their choices on a model of decisions and their potential outcomes that resemble a tree. Logistic regression, or LR, uses a logistic function to model the probability of a binary outcome. Gaussian Naive Bayes (GNB) is a statistical classifier that uses a Gaussian distribution and assumes feature independence. Each algorithm has unique methods, advantages, and disadvantages, suitable for various machine-learning applications and data categories.

In ML, ensemble methods such as bagging, boosting, and voting are effective strategies that use the combined knowledge of several separate models to increase the robustness and performance of the model. By building numerous instances of the same model on various subsets of the training data and combining their predictions, a technique known as “bagging,” short for “Bootstrap Aggregating,” reduces variance and overfitting. In contrast, boosting creates a series of models iteratively, with each new model focusing on fixing the flaws in the previous one to progressively increase prediction accuracy. A balanced majority is produced by voting, whether it be by hard or soft voting, which combines predictions from several models and outputs the most common forecast or average probability, respectively.

## Results

To evaluate the proposed approach, several performance metrics, including training accuracy, test accuracy, precision, recall, F1 score, negative predictive value (NPV), false positive rate (FPR), false discovery rate (FDR), false negative rate (FNR), and Matthews correlation coefficient (MCC) are derived. The results are computed using the values of true positives (TP), true negatives (TN), false positives (FP), and false negatives (FN) from the confusion matrix. In the first experiment, two GNN models, named GAT and GCN, are used with the dataset. It is found that the GCN model outperforms GAT with a test accuracy of 93.23% while the GAT model achieved a test accuracy of 81.23%. Based on this performance, GCN is selected to be optimized with ablation study.

### Ablation study

To improve the performance of the proposed GCN model, eight experiments are carried out and for each experiment the optimal configuration is selected based on accuracy. Table 5 lists the results of eight ablation experiments.

**Table 5.** Results of ablation study.

Convolutional layers			
No.	No. of convolutional layers	Test accuracy (%)	Average time per step (millisecond)
1	3	95.23	128ms
**2**	**2**	**95.24**	**132ms**
3	4	93.73	140ms

Hidden units			
No.	Hidden units	Test accuracy (%)	Average time per step
**1**	**64,64**	**96.21**	**128ms**
2	32,32	84.23	8s

Combination type			
No.	Combination type	Test accuracy (%)	Average time per step
1	concat	87.35	128ms
**2**	**ConvLSTM1D**	**96.21**	**128ms**

Activation function			
No.	Activation function	Test accuracy (%)	Average time per step
**1**	**elu**	**97.73**	**128**
2	relu	87.35	128
3	tanh	91.35	128
4	softmax	96.21	128

Optimizers			
No.	Optimizer	Test accuracy (%)	Average time per step
1	Adam	96.32	128ms
2	Nadam	97.73	128ms
3	Adamax	91.45	128ms
**4**	**SGD**	**96.51**	**128ms**

Learning rates			
No.	Learning rate	Test accuracy (%)	Average time per step
**1**	**0.01**	**97.73**	**128ms**
2	0.001	82.61	20ms
3	0.0001	84.17	20ms
4	0.0007	56.19	6s

Dropout value			
No.	Dropout	Test accuracy (%)	Average time per step
**1**	**0.3**	**98.73**	**128ms**
2	0.4	81.41	72ms
3	0.5	77.95	20ms

Batch size			
No.	Batch size	Test accuracy (%)	Average time per step
1	32	81.48	74ms
**2**	**64**	**98.73**	**7s**
3	128	85.11	128ms

In the first experiment, three configurations are experimented with a different number of convolutional layers. The highest test accuracy of 95.73% is achieved while using four convolutional layers in 140 ms. For a hidden unit size of [64,64], the highest test accuracy of 96.21% is attained. Hence, in the proposed architecture, the number of convolutional layers is three and the hidden unit size is [64,64]. Two combination types are explored. The highest accuracy is obtained for ConvLSTM1D. Employing the activation function ELU, the test accuracy improves from 96.21% to 97.73%. The test accuracy remains does not improve in the next two ablation studies, changing the optimizer and the learning rate. With a dropout rate of 0.3, the accuracy improved to 98.73%, and this performance is maintained using batch size of 64. After completing all the eight ablation studies, the optimal configuration of model architecture is: three convolutional layers, a hidden unit size of [64,64], combination type ConvLSTM1D, activation function ELU, optimizer Nadam, learning rate 0.01, dropout rate 0.3, and batch size 64.

### Performance analysis of the proposed GCN model

**Figure 13.**
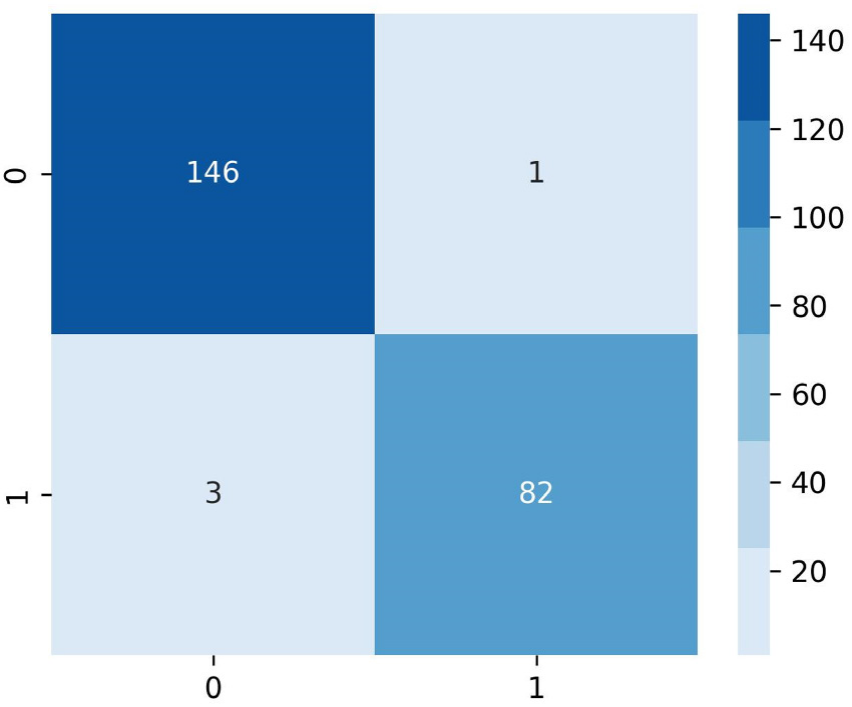
Confusion metrics of the proposed GCN model.

Precision, specificity, sensitivity, NPV, FPR, FDR, FNR, accuracy, F1 score, and MCC are calculated to evaluate the performance of the proposed model. Figure 13 shows the confusion matrix of the model where 0 represents the class benign and 1 represents the class malignant.

Using the values of TP, TN, FP, and FN of the confusion matrix evaluation metrics results are computed. The model performs exceptionally well on a range of evaluation metrics, demonstrating the ability to classify instances correctly. The model can learn from the training dataset with an excellent training accuracy of 99.12%. It remarkably keeps up a high-test accuracy of 98.73%, indicating good adaptation to test data. The model's sensitivity and accuracy, which are essential for evaluating its capacity to identify positive cases and reduce false positives, are 99.32% and 97.99%, respectively. With a notable 98.80% specificity, the model's ability to accurately identify negative cases is highlighted. A fair trade-off between recall and precision is reflected in the F1 score of 98.65%, which is important for classification tasks. Furthermore, the model's ability to handle imbalanced datasets is demonstrated by MCC, which stands at 96.28%. The model is often effective in minimizing prediction mistakes because of its low rates of FPR 0.012%, FDR 0.006%, and FNR 0.02%. Additionally, the model's accuracy in predicting negative cases is demonstrated by its 96.47% NPV. All together, these comprehensive metrics indicate a well-balanced, highly effective model that is appropriate for the present classification task.

**Figure 14.**
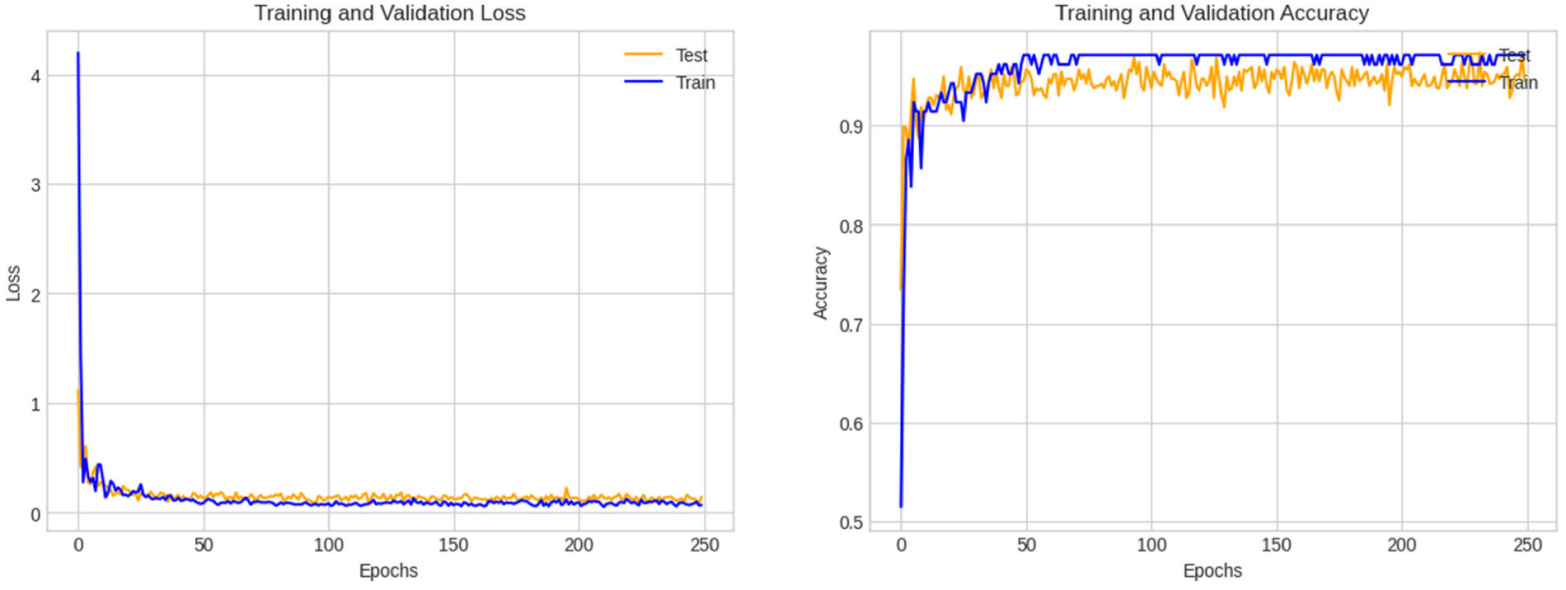
Accuracy and loss curves of our proposed GCN model.

In Figure 14, the loss curve and accuracy curves, during the training period, show minimal variations over the epochs. Though some fluctuation is observed for the validation accuracy and loss curves, there is no significant indication of overfitting. This further validates the robustness of the proposed architecture.

### Evaluating different threshold value of Pearson correlation

This experiment compares the effect of threshold values on the model's performance. This experiment increases accuracy, retains important features, and aids in identifying the optimized threshold value for the graph generation. The results are shown in Table 6.

**Table 6.** Results of the model performance on different threshold value.

*Threshold*	*Test accuracy*	*Total edge number*
*0.15*	95.47	26
***0.20***	**98.73**	**17**
*0.30*	96.51	12
*0.40*	92.71	8

The experiments on at various threshold levels are shown in Table 6. The effects of four different thresholds (0.15, 0.20, 0.30, and 0.40) on test accuracy and the total number of edges found are investigated. Notably, 98.73% test accuracy was achieved with a threshold of 0.20, which also detected 17 edges. This implies that accuracy and feature inclusion have been well-balanced. The accuracy dropped somewhat to 95.47% but the total edge count climbed to 26 when the threshold was lowered to 0.15. On the other hand, greater thresholds of 0.30 and 0.40 resulted in a decrease in accuracy along with a reduction in the overall edge count to 12 and 8, respectively.

### K-fold cross-validation

**Figure 15.**
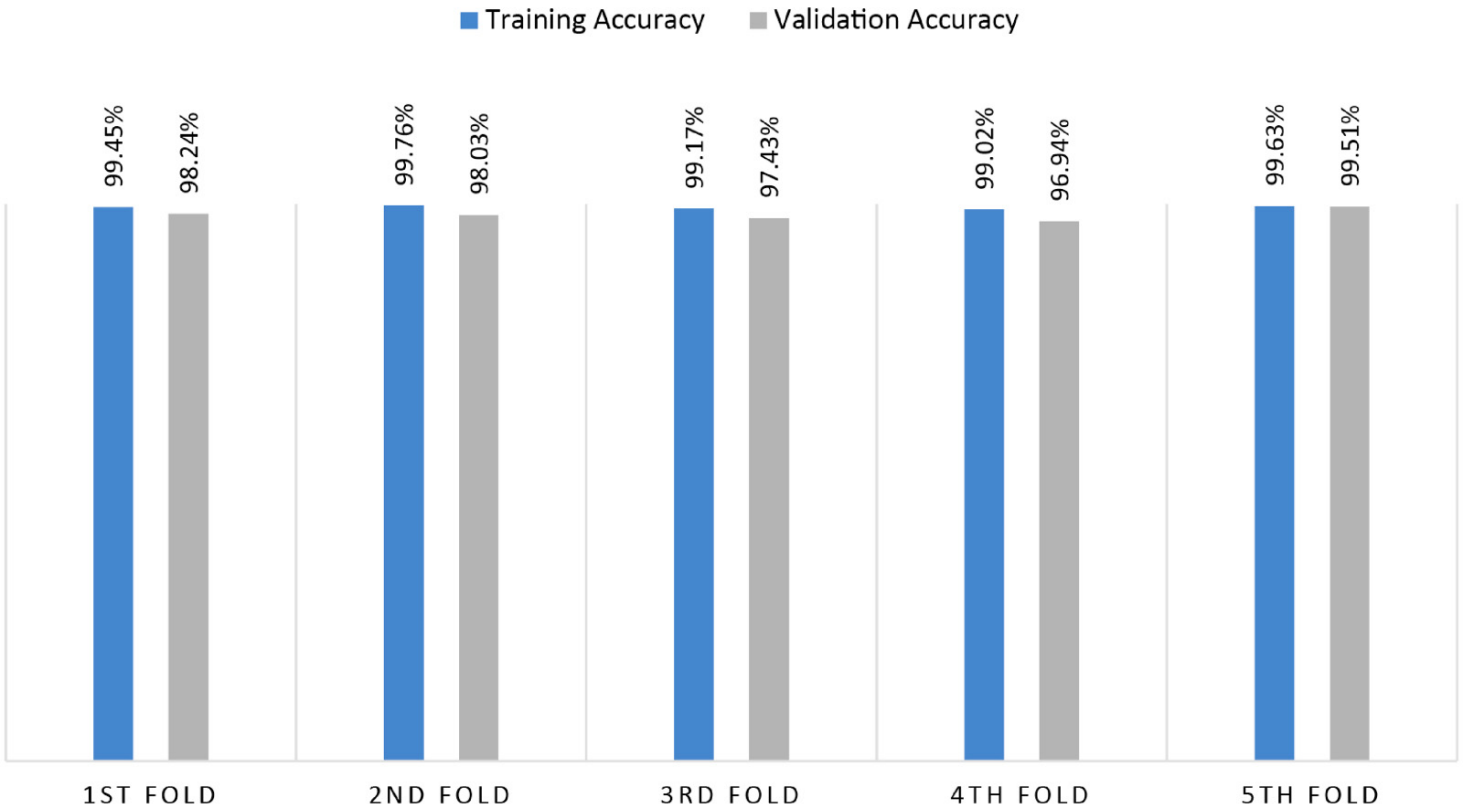
Performance of the model for 5-fold cross-validation.

To further assess overfitting issues and performance consistency of the proposed GCN architecture, K-fold cross-validation is performed. This is an effective approach for assessing how well a model performs on a dataset. In this case, 5-fold validation is considered. It involves splitting the dataset into five folds or subsets of equal size. The model is subsequently trained and assessed five times, using a new fold as the validation set and the other four folds for training. In Figure 15, the performance of 5-fold validation is illustrated.

From Figure 15, it can be observed that across all folds, the validation accuracy is above 96% and the training accuracy is above 98%. The fifth fold yields the highest validation accuracy, of 98.51%, and the fourth fold yields the lowest validation accuracy, of 96.94%. No major gap is found between the training and the validation accuracy for any of the folds, which validates the absence of overfitting problems. The model can provide validation accuracies close to the highest accuracy for each of the folds which demonstrate the performance consistency of the model remarkably.

### Performance comparison analysis

The performance of the proposed approach is compared from two perspectives: feature-based and image-based. In feature-based comparison, GCN model is compared to ML, ensemble ML, GAT and 1D CNN model with the seven extracted features. Additionally, image-based comparison is carried out with transfer learning models.

#### Comparison with 1D CNN and GAT model

Table 7 shows a performance comparison of the GCN model with a 1D CNN model and GAT model in terms of test accuracy, sensitivity, precision, specificity, NPV, FPR, FDR, FNR, F1 score, and MCC.

**Table 7.** Performance comparison of the GCN model with the CNN model and GAT model.

*Performance metrics*	*1D CNN model (%)*	*GAT*	*GCN* m*odel (%)*
*Test accuracy*	91.20	81.23	98.73
*Sensitivity*	97.99	80.13	97.99
*Precision*	90.12	89.63	99.32
*Specificity*	76.12	83.13	98.80
*NPV*	94.44	69.70	96.47
*FPR*	23.88	16.87	0.012
*FDR*	0.09	10.37	0.006
*FNR*	0.02	19.87	0.02
*F1 Score*	93.89	84.62	98.65
*MCC*	79.16	61.26	96.28

It can be observed from Table 7 that with the same features, the 1D CNN model provides a test accuracy of 91.20% with the image feature-based input which is approximately 7% lower than the test accuracy of the GCN model. This experiment highlights the superiority of utilizing graph data and employing graph-based classification methods compared to feature-based 1D-CNN. On the other hand, with the same graph input as the GCN model, the GAT model achieves an accuracy of 81.23%. Based on the other performance metrics, the performances of 1D CNN and GAT model are notably less than the GCN model. The superior performance of the GCN model can be attributed to its ability to effectively capture the underlying relationships and dependencies within the graph-based representation of breast ultrasound image features. The graph data structures are particularly beneficial when dealing with small datasets, as they provide an effective meaning of holding the intricate relationships that exist between various data points.^42,43^ The GCN model excels in learning informative node embeddings through a message-passing framework, allowing it to efficiently aggregate and process neighborhood information. While the optimized FFN layer integrated into the GCN architecture further enhances the model's capacity to identify intricate patterns in the handcrafted features extracted from the images. In contrast, the GAT model employs self-attention mechanisms to compute representations of a single sequence, making it suitable for handling inputs of varying sizes while focusing on critical information. However, the objective of this study is to identify the most important features for detecting cancer. Focusing on medically relevant features that clearly distinguish between benign and malignant cases reduces the complexity of the node information and connection. This streamlined approach minimizes the need for intricate node selection mechanisms, contributing to the GCN model's superior performance in this specific context.

#### Comparison with transfer learning models

**Figure 16.**
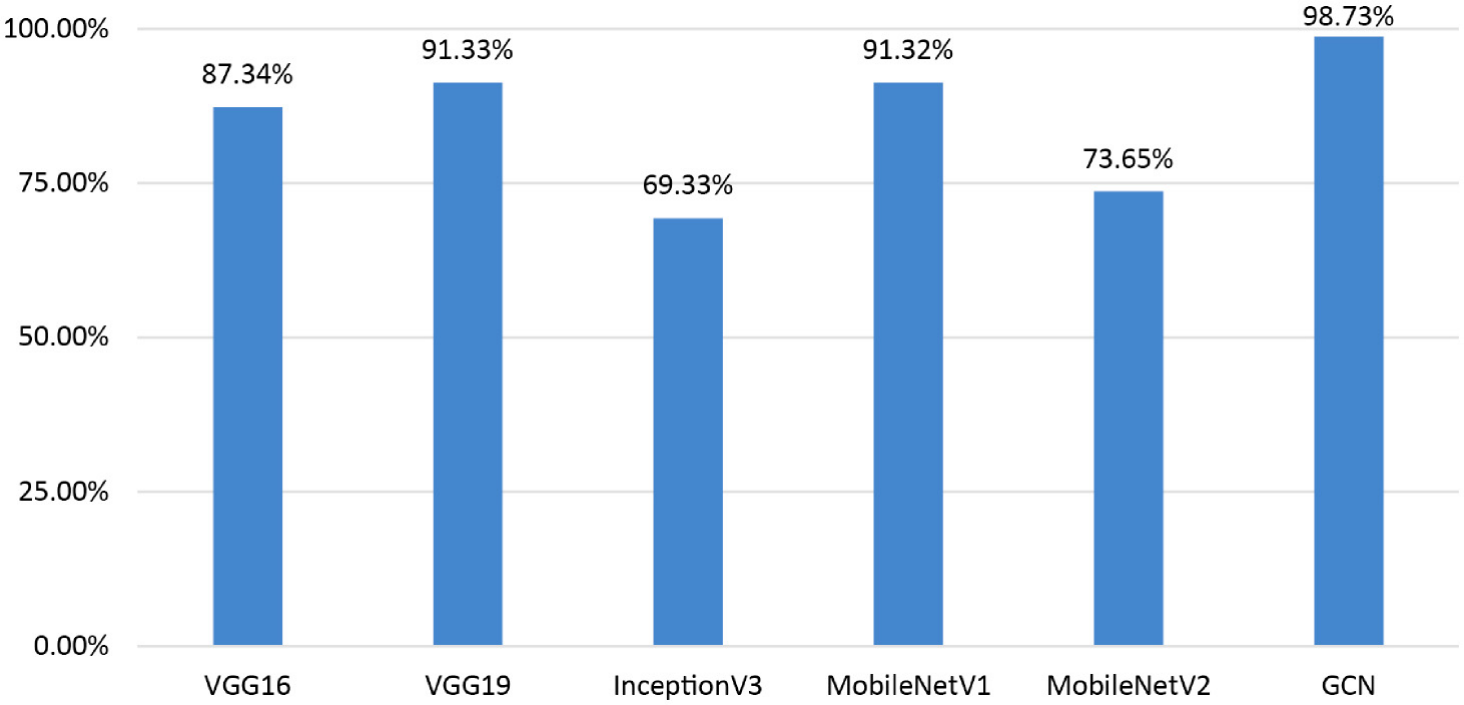
Performance comparison of the GCN with transfer learning models.

Further, a comparative analysis is conducted using traditional transfer learning models in order to thoroughly evaluate the performance of our proposed model. Specifically, the model is trained with ROI images from both benign and malignant cases. The models are VGG16, VGG19, InceptionV3, MobileNetV1, and MobileNetV2. While the primary aim of this study is to leverage clinical biomarkers for the classification task with GCN model, the inclusion of CNN variants plays a crucial role in the performance comparison. The exploration of these variants highlights the limitations of relying solely on image-based features extracted by established pre-trained models, underscoring the importance of focusing on clinically relevant features in medical imaging. Although deep learning models excel at extracting high-level features, the incorporation of GCN addresses the interconnected nature of clinical markers, providing more accurate and interpretable results in breast cancer diagnosis. This comparison analysis shows in Figure 16 that our proposed model exceeds the performance of these well-known transfer learning models.

Our model shows a significant performance improvement when compared to well-known transfer learning models as VGG16 (87.34%), VGG19 (91.33%), InceptionV3 (69.33%), MobileNetV1 (91.32%), and MobileNetV2 (73.65%). It can be observed that using the whole image as input leads to poorer outcome in terms of breast cancer classification with ultrasound images. These findings validate the effectiveness of our method, which not only outperforms well-known architectures but also offers an established framework for reliable and precise health diagnosis. Hence, utilizing clinically significant features to generate graph data can be a more suitable approach. Based on the findings of this performance comparison, it suggested that incorporating the GCN model with graph data demonstrates promising outcomes in the classification of breast ultrasound images.

#### Performance comparison with ML and ensemble ML models

The performance of our proposed model is further compared with the state-of-the-art ML models. This comparison is intended to measure our proposed model's efficacy against existing ML models that are frequently used in medical image analysis. In contrast, ten ML models are compared to our GCN model: the GBC, BC, ETC, RFC, logistic regression (LR), 1D CNN model, GNB, KNN classifier, DTC, SVM, support vector classifier (SVC), and GAT model. Additionally, the performance of GCN model is compared with ensemble ML models. For this comparison BC, boosting classifier, and voting ensemble algorithms are taken. RFC and DTC models are ensembled for BC, Adaboost and GBC are ensembled for Boosting classifier, lastly in voting algorithms LR, DTC, and SVC are ensembled. The results of the comparison are shown in Table 8.

**Table 8.** Comparison with ML and ensemble ML models.

Models	Test accuracy	Ensemble ML models	Test accuracy (%)	Recall (%)	Precision (%)	F1-score (%)	Support (%)
Proposed GCN	98.73%	Bagging classifier	85.32	82.27	82.26	82.66	231
RFC	97.61%						
ETC	61.10%						
GBC	73.15%						
BC	94.95%	Boosting classifier	76.19	76.19	76.26	76.22	
SVM	88.55%						
SVC	88.10%						
KNN	49.54%						
DTC	90.59%	Voting algorithm	75.32	75.32	74.60	74.51	
LR	53.35%						
GNB	47.13%						

In the classification of breast ultrasound images, the proposed GCN network outperforms all existing state-of-the-art models with a test accuracy of 98.73%. Notably, with an accuracy of 94.95%, the RFC also shows good performance, proving its competitiveness in this challenge. With an accuracy of 94.1%, the DTC comes in close second. On the other hand, models with accuracies ranging from 47.13% to 53.35% include KNN, LR, and GNB. In ensemble comparison BC, boosting classifier, and voting algorithms are achieved 85.32%, 76.19%, and 75.32% accordingly. This implies that the proposed GCN is an effective model for classifying breast ultrasound images since it effectively captures complicated relationships within the images by utilizing graph-based representations.

### Experiments with different datasets

The GCN model is further experimented on two different datasets to determine its performance on unseen data. Additionally, the model performance is again analyzed with the merged version of these two datasets. The proposed framework was applied to the first dataset (Dataset A^44^), the second dataset (Dataset B^45^), and the merged dataset (Dataset C) and the outcomes are examined with the evaluation metrics, shown in Tables 9 to 11. The comparative study provides a thorough comprehension of the model's performance in various data scenarios and offers insightful information for the wider implementation of our proposed model. Dataset descriptions are added in Table 12.

**Table 9.** Evaluation metrics performance on BUS dataset.

Performance metrics	Results (%)	Performance metrics	Results (%)
Training accuracy	99.72	NPV	85.26
Test accuracy	91.03	FPR	13.83
Validation accuracy	91.15	FDR	6.06
Sensitivity	93.24	FNR	6.07
Precision	93.69	F1 score	93.46
Specificity	86.17	MCC	79.18

**Table 10.** Evaluation metrics performance on breast-ultrasound-images (Dataset B).

Performance metrics	Results (%)	Performance metrics	Results (%)
Training accuracy	99.72	NPV	89.69
Test accuracy	94.37	FPR	7.07
Validation accuracy	93.92	FDR	3.03
Sensitivity	95.19	FNR	4.07
Precision	96.59	F1 score	95.88
Specificity	92.55	MCC	87.01

**Table 11.** Evaluation metrics performance on merged version of Dataset A & Dataset B (Dataset C).

Performance metrics	Results (%)	Performance metrics	Results (%)
Training accuracy	99.84	NPV	81.14
Test accuracy	89.62	FPR	5.05
Validation accuracy	88.92	FDR	9.07
Sensitivity	90.29	FNR	27.06
Precision	94.17	F1 Score	92.19
Specificity	88.20	MCC	76.89

**Table 12.** Dataset description of Dataset A, Dataset B, and Dataset C.

Dataset	Number of images	
	Benign	Malignant
Dataset A ^44^	1269	606
Dataset B ^45^	431	209
Dataset C (merged of Dataset A & B)	1700	815

The BUS dataset is utilized as Dataset A. Subsequently, a publicly available breast ultrasound images dataset is used from Kaggle as the Dataset B. For Dataset C, Dataset A, which has 1875 images, and Dataset B, which has 640 images, are merged to ensure a more comprehensive representation of variations in imaging conditions which explains the model's adaptability. The evaluation metrics performance for Dataset A is shown in Table 9.

The comprehensive evaluation of GCN model trained on Dataset A is summarized in Table 9. The model performs exceptionally well, as evidenced by its impressive training accuracy of 99.72%, which indicates that it is adept at learning from the training set. The model maintains excellent accuracy levels on the test (91.03%) and validation (91.15%) datasets when its generalization ability is evaluated, indicating a strong capacity to produce accurate predictions on new, unseen cases. Additionally, the model exhibits a well-balanced classification strategy, with a precision (93.69%) indicating its accuracy in positive predictions and a sensitivity (93.24%) indicating its ability to properly identify positive cases. The model's 86.17% specificity highlights how well it can identify negative examples. Remarkably, the F1 score of 93.46% achieves a pleasing mix between sensitivity and precision.

The evaluation metrics performances of GCN model for Dataset B are shown in Table 10.

A thorough summary of the performance indicators for the GCN model trained on Dataset B is given in Table 10. The model demonstrates a high level of efficiency in learning from the training dataset, as evidenced by its remarkable 99.72% training accuracy. The model's impressive test accuracy of 94.37% and validation accuracy of 93.92% demonstrate that it can handle new, unseen data, demonstrating its strong generalization capabilities. With a high sensitivity of 95.19% in classification, the model shows a strong balance, highlighting its accuracy in properly detecting instances that are positive. With a precision of 96.59%, the model's accuracy in positive predictions is as outstanding. Specificity, which gauges how well the model detects negative cases, is a respectable 92.55%.

The evaluation metrics performances are shown in Table 11.

The GCN performance metrics, which were trained on Dataset C, are displayed in Table 11. The model performs exceptionally well throughout training, as demonstrated by its remarkable 99.84% training accuracy. On the other hand, the test accuracy drops to 89.62% when evaluated on new, unseen data, indicating a little decline in generalization performance. Sensitivity stands out at 90.29%, demonstrating the model's capacity to accurately identify positive cases, while the model maintains a comparatively high precision of 94.17%, indicating accuracy in positive predictions. However, a somewhat elevated FNR of 27.06% indicates certain difficulties in identifying every positive instance. At 92.19%, the F1 score—a harmonic means of sensitivity and precision—is impressive and shows a sensible trade-off between these measurements. By evaluating the model's performance and flexibility in a variety of image datasets, this comprehensive study aims to throw insight on the model's adaptation abilities and give a solid foundation for future research.

### Evaluation of the edge-node relationship

Understanding the relationships between data features is a fundamental task in data analysis. In this context, by associating the feature analysis with an equation, we can further validate the observed patterns and gain an insight in the relationships between two features. For each edge of the graph, we have generated an equation in order to assess the correlation among the nodes (features). These equations are based on the equation y=mx+c . Each edge has two features connected to it, and the equation is based on a regression analysis of these features. In Table 13, five edges are listed with their accompanying equation.

**Table 13.** Equations for five edges of the graph.

*No.*	*Source node*	*Target node*	*Linear equation*
*Equation 1*	Solidity	SIFT key points	SIFTKeypoints=−266.12*solidity+274.03
*Equation 2*	Solidity	Width-Height ratio	WHratio=3.17*solidity+(−1.21)
*Equation 3*	Solidity	Ecliptic ratio	ECL ratio = 1.48*Solidity + (−0.48)
*Equation 4*	Solidity	Harris corner	Harris corner = −107.59*Solidity+125.23
*Equation 5*	Solidity	Circularity	Circularity = 1.83*Solidity+(−1.05)

Each equation of Table 13 represents the correlation between two connected nodes. Overall, it is evident that the proposed approach can effectively classify breast cancer with the help of optimal graph data (generated using clinical features) and GCN model.

## Comparison with previous studies

This section includes a comparison table that highlights key information from prior research studies with respect to our proposed methodology. Table 14 provides details regarding authors, publication year, proposed work, and test accuracies.

**Table 14.** Comparison with previous studies.

Authors	Year	Dataset	Methodology/model	Test accuracy
Adyasha Sahu^46^	2024	BUSI, BUS2	Utilized three transfer learning models (AlexNet, ResNet, MobileNetV2) with residual learning.	96.92% with BUSI
Masoumeh Taheri^47^	2024	BUSI	transfer learning to obtain initial features, followed by an Ensemble Meta-Feature Space Generator (EMFSG-Net)	97.75%
Sirjani et al.^48,49^	2023	BUS, UDIAT, BUSI	The InceptionV3 network converted to residual inception.	81.00% with BUSI
Deb et al.^49^	2023	BUSI	An ensemble network based on fuzzy ranks utilizing Xception, VGG-Net, Inception, and DenseNet as foundation learners	85.23%
Byra et al.^50^	2021	BUSI	A transfer learning-based hybrid classifier model incorporating scaling layer.	91.5%.
Luo et al.^51^	2023		A classification method based on spatial attention and cross-semantic human-machine knowledge fusion	91.07%.
Ours		BUSI	GCN model for breast tumor classification	98.73%

Table 14 presents a comprehensive comparison of our proposed study with several prior works in breast cancer classification using ultrasound images. It is observed that the classification accuracies of the prior studies lie between mostly 91% and 97% while for Deb et al. the accuracy was quite lower of 85.23%. In comparison, our study stands out with a remarkable accuracy of 98.73% on the BUSI dataset, surpassing the performance of all other studies mentioned in Table 14. This highlights the superiority of our proposed GCN model for breast tumor classification. Notably, our approach outperforms prior works on the same dataset (BUSI), emphasizing the effectiveness of the GCN model in enhancing the accuracy of breast cancer classification from ultrasound images.

## Addressing the research gap: advancing breast cancer image classification towards radiologist expertise

Many studies have been done in the field of classifying breast cancer, and each one presents distinct approaches developed by different researchers. Looking again at previous research, it is clear that several strategies have been developed to address this problem. Using deep learning techniques, the research by Zhang et al.,^9^ Montaha et al.,^10^ and Kalafi et al.^11^ all present innovative approaches to the classification of breast cancer. Zhang et al.^9^ utilized a variety of architectures, with InceptionV3 being the most successful, to obtain an accuracy of 91.0% while concentrating on transfer learning techniques. In order to improve the classification of breast images into four groups, Montaha et al.^10^ used a customized VGG16 model to obtain an amazing accuracy of 98.02%. They placed a strong emphasis on artifact removal, noise reduction, and image enhancement. In the meantime, an attention mechanism was added by Kalafi et al.^11^ to an enhanced VGG16 architecture along with a novel loss function that combined the logarithm of the hyperbolic cosine loss with binary cross-entropy to produce an accuracy of 93%. In other studies, Moon et al.,^16^ Tanaka et al.,^17^ and Eroglu et al.^12^ used feature extraction methods to categorize breast cancer images. Moreover, innovative approaches for the classification of breast cancer are shown in the research by Rhee et al.,^19^ Pfeifer et al.,^20^ and Chen et al.,^21^ each of which makes use of unique GNN strategies and data representations. Although previous research has made progress in the classification of breast cancer, a more thorough study is still required to create a model that can identify breast cancer images in accordance with radiologist evaluations.

After a comprehensive analysis of previous studies, to the best of our knowledge, such rigorous analysis of the tumor feature from the medical perspective was yet to be explored. The uniqueness of this study lies in the computerized approach of automated feature extraction, which analyzes complex features in a highly effective manner, providing radiologists with valuable assistance. The novelty lies in the approach of considering medical features that have previously been overlooked. By incorporating these clinically significant features, the proposed GCN model focuses on the most discriminative aspects of breast masses, such as tumor shape and orientation. Moreover, achieving a robust performance from a small dataset is quite challenging, which has been addressed in this paper by incorporating a graph-based classification system.

## Discussion & future work

Overall, in real-life situations of manual diagnosis, radiologists face significant challenges in terms of time and effort in accurately assuming shape, alignment, borders, and contiguous tissue parts, as well as identifying classes in ultrasound images. Our automated feature extraction method efficiently analyzes complex features, providing radiologists with valuable assistance. In most cases, the computerized approaches are developed employing some well-known and regular handcrafted features where in this study, after thorough and careful review of medical diagnosis process, the most prominent features are identified and selected. Incorporating seven clinically significant features as clinical biomarkers for classification with the GCN model plays a crucial role. These features exhibit clear distinctions between benign and malignant classes, improving the strength of node information. By prioritizing these aspects, the GCN model learns to capture subtle characteristics necessary for differentiating between benign and malignant tumors. For instance, tumor shape and orientation are well-established diagnostic indications, and their incorporation ensures that the GCN focuses on the most discriminative aspects of breast masses. Moreover, to improve the model's precision, the inclusion of clinically significant markers minimizes the influence of irrelevant or noisy data. Radiologists can save time by automatically extracting features from ultrasound images, which potentially helps to improve the accuracy of breast cancer classification. A thorough comprehension of tumor circumstances is facilitated by the system's capacity to produce organized and comprehensive data on shape and structures. This benefit enhances specialist's findings, enabling quicker, smarter decisions, and improving the accuracy and precision of disease diagnosis in clinical practice.

Our future work will focus on user interface design that serves healthcare professionals, with clearly defined techniques for smooth integration into hospitals and ensure the security to protect patient data. In addition, to improve our knowledge of the system's feasibility and efficacy in actual clinical trials, factors including safety compliance, flexibility, training methods, and cost-benefit analysis will be carefully investigated. Although our model demonstrated impressive accuracy on publicly available datasets, some limitations highlight the difficulties in applying these results to real-world scenarios. One major obstacle is the inherent complexity of analyzing ultrasound images, which is often made harder by the complexities found in clinical situations. Real-world data may contain difficulties due to the variability of patient characteristics, varying imaging equipment, and different protocols. Furthermore, more research and clinical validation are required due to the model's effectiveness in a variety of healthcare facilities and its ability to be generalized across distinct patient information. Breast cancer diagnosis is a dynamic field with issues related to interpretability and ongoing optimization to meet changing standards and new technologies. However, our study offers a promising basis; these shortcomings must be addressed if our automated classification approach is to be successfully incorporated into real-world clinical environments.

## Conclusion

In summary, our study establishes a novel automated approach for the detection of breast cancer through the use of a graph-based representation of crucial clinical information taken from ultrasound images. The radiologists’ preferred clinical markers, such as tumor shape, margin, orientation, surrounding tissues, are represented by computerized features. These features are used to generate a graph where each feature is a graph node and the connection among the features is the graph edge. The test accuracy of the GCN gives an impressive accuracy of 98.73%, indicating that our method is excellent in correctly identifying tumors as benign or malignant. The significance of the proposed model is further highlighted by ablation studies which also improve the performance of model. The performance of the model is compared with a CNN model (using the same features as input), five transfer learning models (using the images as input), and ten state of art ML models also with three ensemble learning methods. The test accuracy of the transfer learning models lies in a range of 69–98% which indicates that extracting clinically relevant features from the images and classifying accordingly can be a better solution. Even with the same features, the CNN model only has a test accuracy of 91.20% which shows that representing optimal features into graph data can be a more effective classification approach. Also, the performance of ML and ensemble ML couldn't cross the accuracy of GCN model. The proposed model also demonstrates its supremacy by effectively classifying breast cancer from three different ultrasound image datasets. To put it simply, our research validates the efficacy of our proposed automated system for classifying breast cancer and highlights the crucial role that well-represented clinical markers contribute to achieving superior diagnostic precision. This combination of cutting-edge computational methods—graph-based representation and GCN, in particular—offers a promising path for future developments in early breast cancer diagnosis.
